# Structure of a photosystem I supercomplex from *Galdieria sulphuraria* close to an ancestral red alga

**DOI:** 10.1126/sciadv.adv7488

**Published:** 2025-05-16

**Authors:** Koji Kato, Minoru Kumazawa, Yoshiki Nakajima, Takehiro Suzuki, Naoshi Dohmae, Jian-Ren Shen, Kentaro Ifuku, Ryo Nagao

**Affiliations:** ^1^ Research Institute for Interdisciplinary Science and Graduate School of Environmental, Life, Natural Science and Technology, Okayama University, Okayama 700-8530, Japan.; ^2^ Graduate School of Agriculture, Kyoto University, Kyoto 606-8502, Japan.; ^3^ Biomolecular Characterization Unit, RIKEN Center for Sustainable Resource Science, Saitama 351-0198, Japan.; ^4^ Faculty of Agriculture, Shizuoka University, Shizuoka 422-8529, Japan.

## Abstract

Red algae exhibit unique photosynthetic adaptations, characterized by photosystem I (PSI) supercomplexes containing light-harvesting complexes (LHCs), forming PSI-LHCI supercomplexes. In this study, we solved the PSI-LHCI structure of *Galdieria sulphuraria* NIES-3638 at 2.19-angstrom resolution using cryo–electron microscopy, revealing a PSI monomer core associated with seven LHCI subunits. Structural analysis uncovered the absence of phylloquinones, the common secondary electron acceptor in PSI of photosynthetic organisms, suggesting adaptation to a benzoquinone-like molecule. Phylogenetic analysis suggests that *G. sulphuraria* retains traits characteristic of an ancestral red alga, including distinctive LHCI binding and interaction patterns. Variations in LHCI composition and interactions across red algae, particularly in red-lineage chlorophyll *a*/*b*–binding–like protein and red algal LHCs, highlight evolutionary divergence and specialization. These findings not only deepen our understanding of red algal PSI-LHCI diversification but also enable us to predict features of an ancestral red algal PSI-LHCI supercomplex, providing a framework to explore evolutionary adaptations from an ancestral red alga.

## Teaser

Structure of *Galdieria sulphuraria* PSI-LHCI unveils ancestral traits, including different quinones and LHCI organizations.

## INTRODUCTION

Oxygenic photosynthesis in cyanobacteria, algae, and land plants harnesses solar energy, converting it into chemical energy with the concomitant production of molecular oxygen (*1*). This light-driven energy conversion is facilitated by two multisubunit membrane protein complexes, photosystem I (PSI) and PSII, which orchestrate light absorption, charge separation, and electron-transfer processes (*2*–*5*). Various light-harvesting antenna subunits bind to the PSI and PSII cores, optimize the capture of solar energy, and facilitate the transfer of excitation energy to the photosystems (*1*). These antennae exhibit remarkable diversity across photosynthetic organisms in terms of protein sequences and pigment compositions, and are broadly categorized into membrane-bound and water-soluble proteins (*1*).

The membrane-bound antennae predominantly comprise the light-harvesting complex (LHC) protein superfamily (*6*, *7*), which absorbs solar energy through chlorophylls (Chls) and carotenoids (Cars). Variations in the number and types of Chls and Cars among LHCs contribute to the color diversity observed in photosynthetic organisms, which are broadly classified into green and red lineages (*8*). The green lineage includes organisms such as green algae and land plants, while the red lineage encompasses red algae, diatoms, haptophytes, cryptophytes, and dinoflagellates (*8*). LHCs specific to PSI (LHCIs) associate with eukaryotic PSI monomers to form the PSI-LHCI supercomplexes (*9*, *10*), whose structures have been elucidated in various eukaryotic species through cryo–electron microscopy (cryo-EM) (*9*, *10*).

The red-lineage algae have evolved from red algae (*11*), and the number of LHCIs, as well as their protein and pigment compositions, exhibits considerable variability (*10*, *12*–*14*). Red algae constitute a distinct photosynthetic lineage, encompassing both unicellular and large multicellular taxa (*15*), and serve as an evolutionary intermediate between cyanobacteria and red-lineage eukaryotic algae through a serial endosymbiosis event (*8*, *11*). Recent structural studies have revealed that the red alga *Cyanidioschyzon merolae*, a member of Cyanidiophyceae (Cyanidiophytina) (*16*–*19*), features PSI-LHCI structures containing three to five LHCI subunits (*20*, *21*). Similarly, the red alga *Cyanidium caldarium*, a member of Cyanidiophyceae (*16*–*19*), has a PSI-LHCI structure containing five LHCI subunits, as observed in the strain NIES-2137 (*22*). In contrast, the red alga *Porphyridium purpureum*, belonging to Porphyridiophyceae, an order included in Rhodophytina, which is a group different from Cyanidiophyceae, displays a PSI-LHCI structure with seven LHCI subunits and a red-lineage Chl *a*/*b*–binding–like protein (RedCAP) (*23*), which is part of the LHC protein superfamily (*6*, *7*). These observations underscore notable differences in both the number and binding sites of LHCIs across these three red algal species.

The red alga *Galdieria sulphuraria* belongs to Galdieriales, an order that likely retains more ancestral red algal traits than other Cyanidiophyceae, such as Cavernulicolales, Cyanidiales, and Cyanidioschyzonales (*19*). Phylogenetic analysis robustly supported that Galdieriales diverged earliest, followed by Cavernulicolales, which diverged before Cyanidiales and Cyanidioschyzonales (*19*). Furthermore, Cyanidiales and Cyanidioschyzonales have undergone substantial genome reduction (*24*). The shared characteristics of Galdieriales with Rhodophytina—the other group of red algae outside of Cyanidiophyceae—can provide key information on the trajectory of early red algal evolution. In particular, the structural arrangement of LHC around PSI in Galdieriales is predicted to provide insights into the assemblage of the ancestral red algal structure (*25*). While negative-stain electron microscopy has suggested features of the PSI-LHCI supercomplexes in *G. sulphuraria* (*26*), the overall structure has yet to be determined.

In this study, we solved a PSI-LHCI structure of *G. sulphuraria* NIES-3638 at a resolution of 2.19 Å by cryo-EM single-particle analysis. The structure shows a PSI monomer core and seven LHCI subunits. On the basis of the structural and phylogenetic analyses of LHCIs, we propose an ancestral red algal PSI-LHCI structure and provide evolutionary insights into the conservation and diversity of red-lineage LHCIs.

## RESULTS

### Overall structure of the *G. sulphuraria* PSI-LHCI supercomplex

**Fig. 1.**
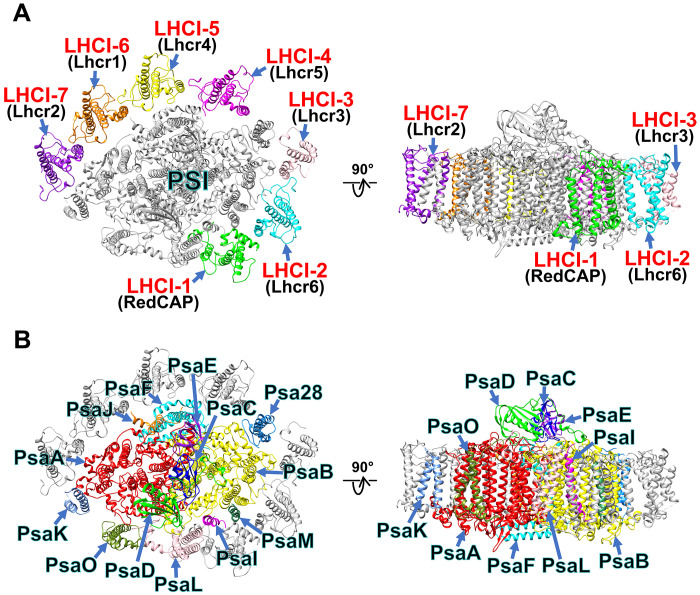
Overall structure of the PSI-LHCI supercomplex from *G. sulphuraria*. Structures are viewed from the stromal side (left) and the direction perpendicular to the membrane normal (right). Only protein structures are shown, and cofactors are omitted for clarity. The LHCI (**A**) and PSI core (**B**) subunits are labeled and colored differently. The seven LHCI subunits are labeled as LHCI-1 to LHCI-7 (red), with their gene products indicated in parentheses (black) in (A). “Lhcr” stands for the light-harvesting complex subfamily originally found in red algae, and "RedCAP" for the red lineage chlorophyll *a*/*b*-binding-like protein.

The PSI-LHCI supercomplexes were purified from the red alga *G. sulphuraria* NIES-3638, and their biochemical and spectroscopic properties have been characterized previously (*27*). Cryo-EM images were obtained using a JEOL CRYO ARM 300 electron microscope operated at 300 kV, yielding a final cryo-EM map at 2.19-Å resolution with C1 symmetry (figs. S1 and S2 and table S1). The atomic model of PSI-LHCI was built on the basis of this cryo-EM map (see Materials and Methods, fig. S2, and tables S1 to S3). The structure reveals a monomeric PSI core bound with seven LHCI subunits (Fig. 1, A and B). The seven LHCI subunits are designated LHCI-1 to LHCI-7 (Fig. 1A) following the naming convention established for LHCI subunits in the PSI-LHCI structure of *C*. *caldarium* RK-1 (NIES-2137) (*22*); among which, the names and binding sites of LHCI-1 and LHCI-2 are conserved between the *G. sulphuraria* and *C. caldarium* PSI-LHCI structures. The PSI core of *G. sulphuraria* includes 99 Chls *a*, 22 β-carotenes (BCRs), 5 zeaxanthins (ZXTs), 3 [4Fe-4S] clusters, 2 unknown quinone molecules, and 6 lipid molecules, whereas the 7 LHCI subunits include 69 Chls *a*, 25 ZXTs, 4 β-cryptoxanthins (BCXs), 2 BCRs, and 5 lipid molecules (table S3).

### Structure of the *G. sulphuraria* PSI core

The PSI core comprises 13 subunits: PsaA, PsaB, PsaC, PsaD, PsaE, PsaF, PsaI, PsaJ, PsaK, PsaL, PsaM, PsaO, and Psa28 (Fig. 1B). The arrangement of these subunits, excluding Psa28, is conserved among the three species of *G. sulphuraria*, *C. merolae*, and *C. caldarium* in Cyanidiophyceae (*21*, *22*). Notably, all 13 subunits in the *G. sulphuraria* PSI-LHCI (GsPSI-LHCI) structure closely match those in the PSI-LHCI structure of *P. purpureum* in Porphyridiophyceae of Rhodophytina (*23*). Encoded by the nuclear gene *Psa28*, the Psa28 subunit features two transmembrane helices that interact with PsaB. Psa28 was initially identified in the diatom *Chaetoceros gracilis* (*28*, *29*) and has also been observed in the PSI core of various red-lineage algae, including the rhodophyte *P. purpureum* (*23*), cryptophytes *Chroomonas placoidea* (*30*), *Rhodomonas salina* (*31*), dinoflagellates *Symbiodinium* sp. (*32*, *33*), and *Amphidinium carterae* (*33*). Psa28 is also referred to as PsaR; however, the name of Psa28 was adopted here on the basis of the historical background of nomenclature of PSI proteins, as explained in our recent study (*34*).

### Molecular evolution and significance of Psa28

Despite the widespread presence of Psa28 among red-lineage algae, a comprehensive phylogenetic analysis of Psa28 is yet to be conducted. Here, we address this gap by collecting Psa28 sequences from a broad range of red-lineage algae. Psa28 is conserved in the Galdieriales of Cyanidiophyceae and in Rhodophytina, which includes *P. purpureum*. However, Psa28 is absent in Cyanidiales (including *C. caldarium*) and Cyanidioschyzonales (including *C. merolae*) within Cyanidiophyceae, while the presence of Psa28 in Cavernulicolales, an order of Cyanidiophyceae, remains uncertain because of the lack of reported nuclear genome data (*19*).

In the secondary endosymbiotic algae of red lineage, Psa28 is present in cryptophytes, stramenopiles (including diatoms), haptophytes, and dinoflagellates. To identify conserved motifs, we aligned these Psa28 sequences, which reveal that while the two transmembrane helices of Psa28 are highly conserved, two regions display taxon-specific variations, namely, (i) the N-terminal region interacting with PsaB (fig. S3, A and B) and (ii) the structurally diverse lumenal region (fig. S3, C to E). In the N-terminal region, T52, W54, and G56 of *G. sulphuraria* Psa28 interact with E303, A307, and R309 of PsaB at distances of 2.6 to 3.0 Å, respectively (fig. S3A). These residues of Psa28 are conserved not only in other Galdieriales species but also in some Rhodophytina species, e.g., *Gracilariopsis chorda*, *Porphyra umbilicalis*, *Porphyra purpurea*, *Madagascaria erythrocladioides*, and *Rhodella maculata* (fig. S3B), but not in *P. purpureum*, which would use other residues for PsaB-Psa28 interactions. However, considering the conservation between *G. sulphuraria* and some Rhodophytina species, the PsaB-Psa28 interaction in *P. purpureum* may be acquired secondarily. Therefore, it is reasonable to assume that Psa28 is bound to PSI via PsaB, as it was already present in their last common ancestor and has been vertically inherited to Galdieriales and Rhodophytina. Notably, diatoms and Bolidophyceae (synonymously, Parmales) within stramenopiles have a unique protein motif in the lumenal loop region of Psa28 (a green square in fig. S3C), whereas cryptophytes lack both of this motif and the additional residues corresponding to PLTV present in *G. sulphuraria* (a red square in fig. S3C).

**Fig. 2.**
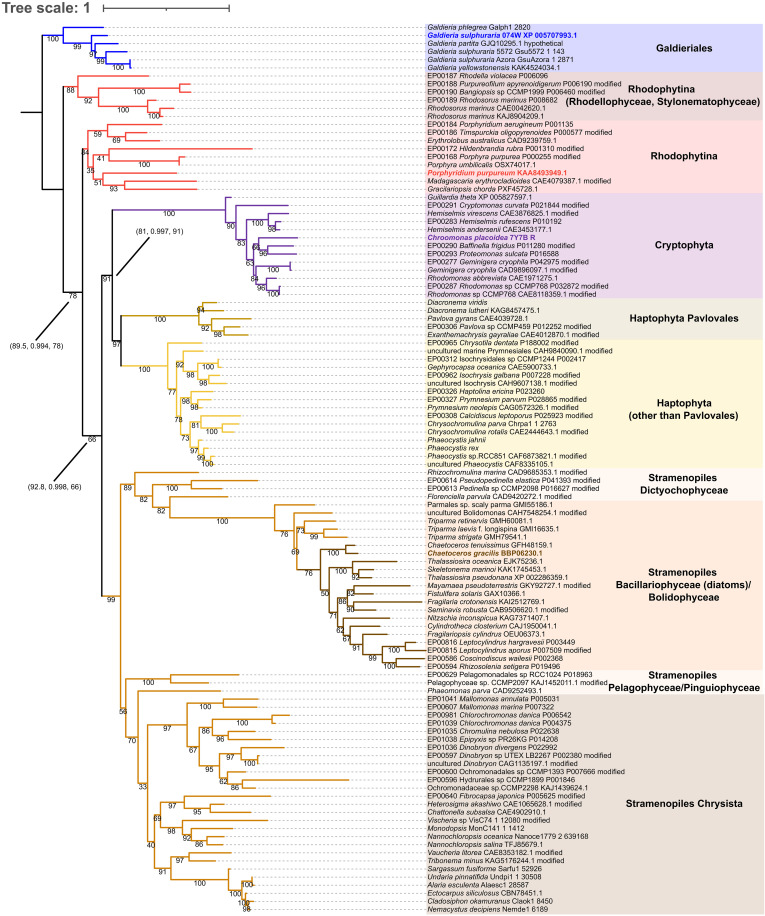
Molecular phylogenetic tree of Psa28. The phylogenetic tree was inferred by IQ-TREE 2 and rooted at the out-group of the Galdieriales RedCAP clade based on the species tree of red algae (*19*, *71*). Numbers on nodes are ultrafast bootstrap support (%). Numbers in parentheses are SH-like approximate likelihood ratio test (SH-aLRT) support (%), aBayes support, and ultrafast bootstrap support (%) values, respectively.

We performed a molecular phylogenetic analysis using Psa28 sequences from a broad range of lineages (Fig. 2), excluding dinoflagellate sequences due to low similarity. The Psa28 clades of the secondary endosymbiotic algae of red lineage are nested within the Rhodophytina clade, forming a monophyletic group. In this large clade, haptophyte and cryptophyte Psa28 sequences also form a monophyletic group, with an ultrafast bootstrap value of 91%. Similarly, Psa28 sequences of heterokonts (photosynthetic stramenopiles) show strong monophyly, with a 99% bootstrap value. Unlike in the RedCAP phylogeny (*25*), however, heterokonts and haptophytes do not form a monophyletic group in the Psa28 phylogeny.

In the Psa28 phylogeny, the secondary endosymbiotic algae of red lineage branch from the Rhodophytina clade, contrasting with the plastid phylogeny, where red-lineage secondary endosymbiotic algae and Rhodophytina form sister groups (*35*). Nevertheless, the Psa28 phylogenetic tree resembles the plastid genome tree, where each secondary endosymbiotic alga of red lineage, such as cryptophytes and haptophytes, forms monophyletic groups. This topology of the Psa28 phylogenetic tree and the conserved PsaB-Psa28 interaction suggest that Psa28 should be inherited vertically from the common ancestor of red algae to Galdieriales and Rhodophytina and horizontally to the red-lineage secondary endosymbiotic algae from red algae. Despite Psa28 being nuclear-encoded, this resemblance to plastid phylogeny may reflect evolutionary constraints driven by its role as a PSI core protein. Specifically, the strong interactions between Psa28 and the adjacent plastid-encoded protein PsaB (fig. S3A) may act as a selective force, maintaining this phylogenetic pattern. These findings offer insights into the evolution and functional adaptation of Psa28 across diverse red-lineage algae.

### Unique quinone molecules of the *G. sulphuraria* PSI core

**Fig. 3.**
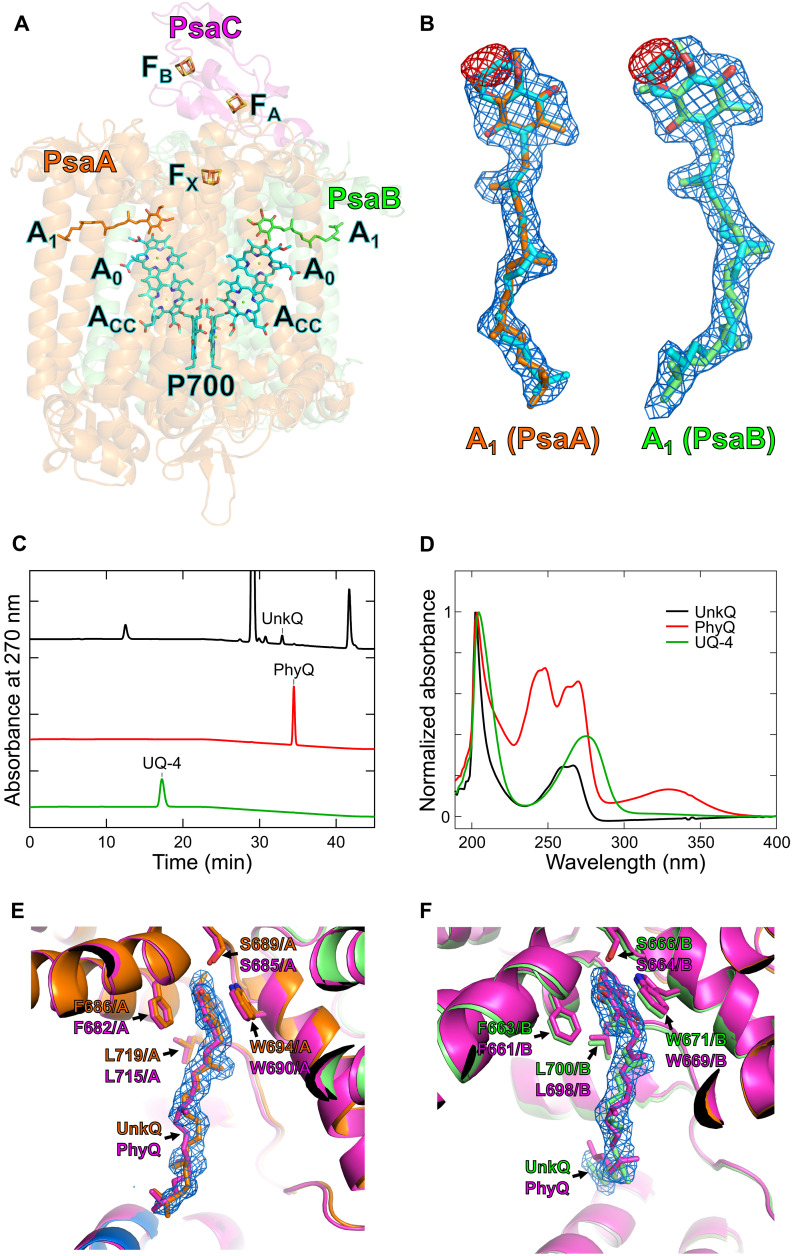
Characteristics of the A_1_ molecules. (**A**) Arrangement of cofactors involved in the electron-transfer reaction of GsPSI-LHCI. P700, special pair Chl; A_CC_, accessory Chl; A_0_, primary electron acceptor; A_1_, secondary electron acceptor; F_X_, F_A_, and F_B_, iron-sulfur clusters. (**B**) Cryo-EM maps and quinone structures (sticks) of the A_1_ molecules. The A_1_ molecules of GsPSI-LHCI were provisionally modeled as ubiquinone-4 in PsaA (orange) and PsaB (green). Two ubiquinone-4 molecules superimposed with phylloquinones (cyan). Red meshes, *F*_o_ − *F*_c_ difference maps (−10 σ) when the A_1_ molecules are modeled as phylloquinones; blue meshes, denoised maps (3 σ). (**C**) HPLC profiles of the *G. sulphuraria* PSI core (black line), phylloquinone (PhyQ; red line), and ubiquinone-4 (UQ-4; green line). The *G. sulphuraria* PSI cores were prepared previously (*27*) and showed a peak of unknown quinone (UnkQ). PhyQ and UQ-4 were purchased (see Materials and Methods). (**D**) Absorption spectra of UnkQ (black line), PhyQ (red line), and UQ-4 (green line). The spectra were measured by a photodiode array detector in the HPLC system and normalized by the maximum peak intensity of each spectrum. Data in [(C) and (D)] are representative of three independent experiments. (**E** and **F**) Structures of PsaA (E) and PsaB (F) that surround the A_1_ molecules. The GsPSI-LHCI structure (orange for PsaA and green for PsaB) was superimposed with the PSI-LHCI structure of *C. caldarium* NIES-2137 [Protein Data Bank (PDB): 8WEY, magenta for both PsaA and PsaB]. Amino acid residues participating in the interactions with the ring of A_1_ are labeled; for example, F686/A and F663/B mean Phe^686^ of PsaA and Phe^663^ of PsaB, respectively.

The electron-transfer chain of the *G. sulphuraria* PSI comprises the special pair Chl P700, accessory Chls A_CC_, primary electron acceptors A_0_, secondary electron acceptors A_1_, and three iron-sulfur clusters F_X_/F_A_/F_B_ (Fig. 3A). Most of the electron-transfer chain components of *G. sulphuraria* are identical to those of *C. caldarium* (*22*); however, the secondary electron acceptors could not be identified as phylloquinones, which are present in PSI of most photosynthetic organisms, including cyanobacteria, algae, and land plants. This discrepancy arises from *F*_o_ − *F*_c_ difference maps (red meshes) of the two A_1_ molecules analyzed by Servalcat, showing negative difference densities at the ring structures between phylloquinone models (cyan sticks) and observed maps (blue meshes) (Fig. 3B). The details of how the quinone models fit the maps are summarized in fig. S4. Ubiquinone-4 was well fitted into the maps corresponding to the two A_1_ molecules, with both the two methoxy groups (black arrows) and a methyl group (red arrows) within their ring structures being clearly resolved (fig. S4, A and B). However, phylloquinone did not fit into the maps at the positions indicated by black arrows (fig. S4, C and D), with apparent steric clashes between the models and the maps. Plastoquinone-9, which is bound to the quinone sites in PSII, lacked a corresponding structural feature in the models at the positions indicated by red arrows (fig. S4, E and F), preventing a satisfactory fit into the maps. High-performance liquid chromatography (HPLC) analysis showed no apparent phylloquinone peak in the PSI core of *G. sulphuraria* (black line in Fig. 3C), with reference to the HPLC chromatogram of pure phylloquinone (red line in Fig. 3C). Similarly, ubiquinone-4 was undetected in the PSI core compared with the reference (green line in Fig. 3C). Instead, a peak for an unidentified quinone appeared at a retention time of 32.9 min (black line in Fig. 3C). The absorption spectrum of this unknown quinone differed from those of phylloquinone and ubiquinone-4 (Fig. 3D) but was more similar to that of the ubiquinone-4 than that of the phylloquinone. The other detectable peaks corresponded to pigments derived from Chls or Cars (fig. S5). On the basis of these findings, we provisionally modeled the two A_1_ molecules as ubiquinone-4, which fit the cryo-EM map, including its ring and side chain (Fig. 3, A and B, and fig. S4).

Both oxygenic and anoxygenic photosynthetic organisms typically use naphthoquinones such as phylloquinone or structurally similar menaquinone-4 as A_1_ molecules in PSI and PSI-type reaction centers (*36*). Green plants use phylloquinone as A_1_ in PSI, as do most cyanobacteria and the red alga *P. purpureum* (*37*, *38*). By contrast, the cyanobacterium *Gloeobacter violaceus*, the red alga *C*. *caldarium* IAM R-11 (NIES-2137), and the diatom *C*. *gracilis* have been reported to use menaquinone-4 as A_1_ (*39*–*41*). Phylloquinone and menaquinone-4, though differing in their side chains, share a naphthoquinone core and exhibit similar electrochemical properties. In cryo-EM structural analyses, distinguishing phylloquinone from menaquinone-4 is highly challenging, and the presence of phylloquinone cannot be excluded in the PSI cores of *C. caldarium* NIES-2137 and *C. gracilis*; consequently, phylloquinone has been modeled in the structures of *C. caldarium* NIES-2137 PSI-LHCI and *C. gracilis* PSI-FCPI (*22*, *28*, *29*). Ubiquinone, with its benzoquinone structure commonly found in respiratory chains, has different electrochemical properties, including a higher redox potential than naphthoquinones (*42*), making it less ideal as an A_1_ electron acceptor. In addition, ubiquinone-4 was not found in the PSI structures from various photosynthetic organisms (*9*, *10*, *43*). Therefore, it is interesting how the benzoquinone-like compound observed in the *G. sulphuraria* PSI may exhibit similar redox potential to phylloquinone and menaquinone-4.

Notably, the surrounding environment of A_1_, including protein structures and amino acid residues of PsaA and PsaB in the PSI-LHCI structure of *G. sulphuraria*, is nearly identical to that of *C. caldarium* NIES-2137 (Fig. 3, E and F, and figs. S6 and S7). The amino acid sequences of PsaA and PsaB had similarities of 94 and 92%, respectively, between *G. sulphuraria* and *C. caldarium* NIES-2137 (figs. S6 and S7). The side chain length of the unknown quinone modeled as ubiquinone-4 matches that of the phylloquinone, and they differ only in their ring structures (Fig. 3B). This suggests that phylloquinone- and ubiquinone-4–like molecules can bind to the A_1_ sites irrespective of their differences in ring structures, as no distinctive cavities exist in the protein environment surrounding the A_1_ ring structures (Fig. 3, E and F).

The presence of different quinones at the A_1_ sites in *G. sulphuraria* may provide insights into the origin of quinones in the red algal PSI cores. The cluster of the menaquinone biosynthesis genes in the plastid genome in Cyanidiophyceae is suggested to be acquired through horizontal gene transfer from Chlamydiae, with the exception of Galdieriales (*19*). However, on the basis of current data, the origin and biosynthesis of the benzoquinone-like compound in GsPSI-LHCI remain uncertain. Further investigation into the chemical identity and biosynthetic pathways of this benzoquinone-like compound is necessary to better understand the diversity and origins of A_1_ in red algae and other photosynthetic organisms.

### Pigment composition of the *G. sulphuraria* PSI core

The number and arrangement of Chls within the PSI core in the GsPSI-LHCI structure (fig. S8A) closely resemble those in the *C. caldarium* PSI-LHCI structure (*22*). However, Chl a102 of PsaI and Chl a201 of Psa28 are unique to the GsPSI-LHCI structure. In contrast, the Car composition of PSI in *G. sulphuraria* (fig. S8B) differs from that in *C. caldarium* (*22*), with ZXT853 of PsaB, ZXT206/ZXT207 of PsaO, and BCR202/ZXT203 of Psa28 found only in *G. sulphuraria*. BCR304 of PsaF in *G. sulphuraria* is replaced by ZXT205 of PsaF in *C. caldarium*.

### Structure of the *G. sulphuraria* LHCIs

The seven LHCI subunits in the PSI-LHCI structure were identified as the products of seven genes: *RedCAP*, *Lhcr6*, *Lhcr3*, *Lhcr5*, *Lhcr4*, *Lhcr1*, and *Lhcr2*, at the sites of LHCI-1 to LHCI-7, respectively (Fig. 1A). These gene names are consistent with those established in previous studies (*27*, *44*, *45*). Notably, RedCAP is distinct from the LHC protein family, although it is grouped within the LHC protein superfamily (*6*, *7*). Each LHCI subunit was identified on the basis of their characteristic amino acid residues on the cryo-EM map, specifically: L137/V139/I141 in LHCI-1, I129/F131/S133 in LHCI-2, F143/L145/A147 in LHCI-3, L132/L134/L136 in LHCI-4, F137/F139/G141 in LHCI-5, L212/F214/I216 in LHCI-6, and V144/W146/S148 in LHCI-7 (fig. S9, A to H). In addition, *G. sulphuraria* has two copies of Lhcr1 and Lhcr3 (fig. S9, I and J), among which the two Lhcr1s differ only in one residue, and the two Lhcr3s have completely the same sequence, so these have no impact on model building in this study. The root mean square deviations (RMSDs) between the structures of LHCI-4 and the other six LHCIs range from 0.99 to 3.35 Å, with LHCI-1 exhibiting a notably higher RMSD compared to the other LHCIs, reflecting its structural distinctiveness (table S4).

Each LHCI subunit contains several Chl and Car molecules, namely, 8 Chls *a*/6 ZXTs/1 BCX in LHCI-1, 10 Chls *a*/3 ZXTs in LHCI-2, 6 Chls *a*/1 ZXT in LHCI-3, 11 Chls *a*/3 ZXTs/1 BCR/1 BCX in LHCI-4, 11 Chls *a*/3 ZXTs/1 BCR/1 BCX in LHCI-5, 12 Chls *a*/4 ZXTs/1 BCX in LHCI-6, and 11 Chls *a*/5 ZXTs in LHCI-7 (fig. S10 and table S3). The axial ligands for the central Mg atoms of Chls in each LHCI subunit are provided by amino acid residues, as well as water and lipid molecules (table S5). Potential excitation-energy-transfer pathways from the antenna subunits to the PSI core were proposed on the basis of close physical interactions among Chls, specifically between LHCI-1 and PsaI (a), LHCI-2 and PsaB (b), LHCI-3 and PsaB (c), LHCI-4 and Psa28/PsaF (d/e), LHCI-5 and PsaJ (f), LHCI-6 and PsaA (g), and LHCI-7 and PsaK (h) (fig. S11).

Among these LHCI subunits, LHCI-6 is the gene product of *Lhcr1*. Our previous study showed that the protein band of Lhcr1 in SDS–polyacrylamide gel electrophoresis appeared at a position with a molecular weight near 29.0 kDa, which is higher than the typical molecular weight range for LHC proteins (14 to 20 kDa) (*27*). The GsPSI-LHCI structure shows that the LHCI-6 subunit starts from K119 (fig. S12), and its structure with three transmembrane helices is virtually identical to the other six LHCI structures (fig. S10). These findings suggest that the N-terminal extension up to K119 is disordered in the GsPSI-LHCI structure and, hence, not visible in the cryo-EM map.

### Structural characteristics of LHCI-1 (RedCAP)

**Fig. 4.**
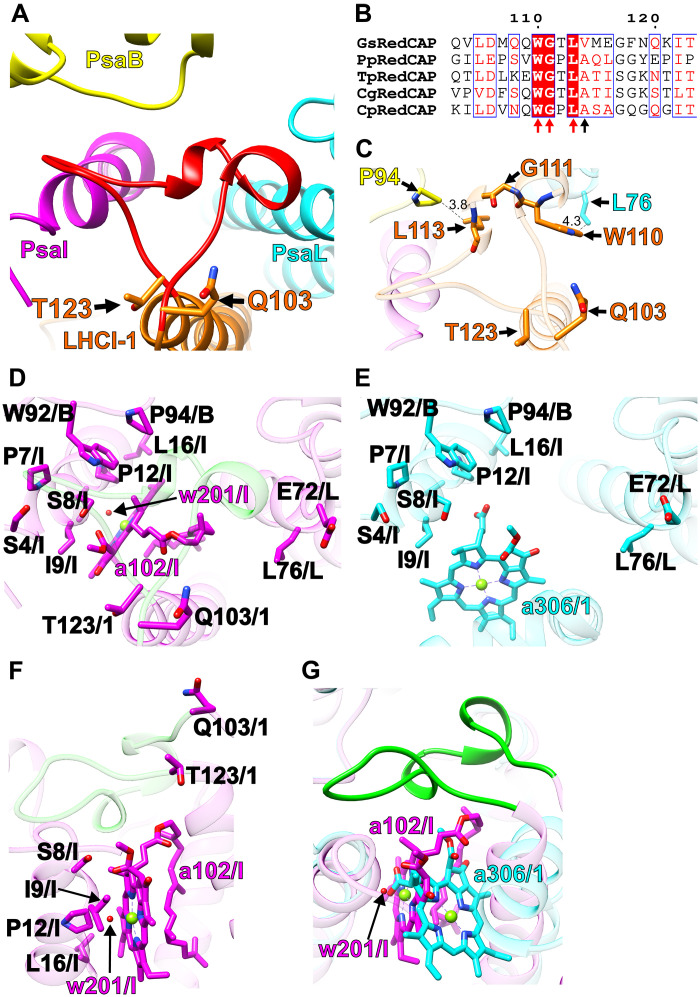
Structural characteristics of RedCAP. (**A**) Protein arrangement of LHCI-1 (RedCAP; orange) with PsaB (yellow), PsaI (magenta), and PsaL (cyan) viewed from the lumenal side. The Q103-T123 loop of RedCAP is highlighted by red. (**B**) Multiple sequence alignment of RedCAP of *G. sulphuraria* NIES-3638 (GsRedCAP) with those of *P. purpureum* UTEX 2757 (PpRedCAP), *T. pseudonana* CCMP1335 (TpRedCAP), *C. gracilis* UTEX LB 2658 (CgRedCAP), and *C. placoidea* T11 (CpRedCAP) using ClustalW (www.genome.jp/tools-bin/clustalw) and ESPript (https://espript.ibcp.fr/ESPript/cgi-bin/ESPript.cgi). The Q103-T123 region was extracted from the whole alignment (fig. S13). Characteristic amino acid residues in the Q103-T123 loop are indicated by red and black arrows. (**C**) Protein-protein interactions of LHCI-1 with PsaL and PsaB. Interactions are indicated by dashed lines, and the numbers are distances in angstrom. Amino acid residues participating in the interactions are labeled. Each color corresponds to that in (A). (**D** to **G**) Arrangements of proteins and cofactors around the Q103-T123 loop in the GsPSI-LHCI structure [magenta in (D), (F), and (G)] and around the corresponding region in the *C. caldarium* PSI-LHCI structures (PDB: 8WEY) [cyan in (E) and (G)]. Superposition of the PSI-LHCI structures between *G. sulphuraria* and *C. caldarium* (G). The Q103-T123 loop is highlighted by green in each panel. Amino acid residues and cofactors are labeled; for example, W92/B means Trp^92^ of PsaB. B, PsaB; I, PsaI; L, PsaL; 1, LHCI-1. The Chl and water molecules are labeled: a102/I for Chl102 of PsaI, w201/I for water201 of PsaI, and a306/1 for Chl306 of LHCI-1, which are highlighted by magenta or cyan in each PSI-LHCI.

The *G. sulphuraria* LHCI-1 was identified as RedCAP, a protein with unique structural features distinct from the LHC protein family (*6*, *7*). RedCAP has been found at the LHCI-1 site in the PSI-LHCI structures of various red-lineage algae (*34*). The characteristic structure of RedCAP is largely defined by a loop region inserted into a cavity formed by PsaB, PsaI, and PsaL at the lumenal side, as seen in the PSI-FCPI structure of the diatom *Thalassiosira pseudonana* (*34*). This distinctive loop, spanning residues Q103-T123 (Fig. 4A), is also present in the GsPSI-LHCI structure. Multiple sequence alignments of RedCAP show conserved Trp, Gly, and Leu residues (red arrows in Fig. 4B and fig. S13), which are part of the Q103-T123 loop in the *G. sulphuraria* RedCAP. We recently hypothesized that the protein motif (xWGxLAxx) plays a key role in RedCAP binding to PSI in the red-lineage algae (*34*); however, the *G. sulphuraria* RedCAP has Val instead of Ala in this loop (a black arrow in Fig. 4B), suggesting that a modified motif (xWGxLxxx) may still support the association of RedCAP with PSI (Fig. 4C).

It is noteworthy that RedCAP is present in *G. sulphuraria* NIES-3638 but absent in *C. caldarium* NIES-2137 and *C. merolae* (*20*–*22*). These species belong to the monophyletic Cyanidiophyceae (*6*, *7*, *19*, *35*) distinct from Rhodophytina (*19*). In *C. caldarium* NIES-2137 and *C. merolae*, the LHCI-1 site is occupied by Lhcr1 (*21*, *22*). We compared the structural environment surrounding the Q103-T123 loop of LHCI-1 in the GsPSI-LHCI with the corresponding region in *C. caldarium* (Fig. 4, D and E). The protein conformations of PsaB, PsaI, and PsaL around the Q103-T123 loop (green cartoon) in the GsPSI-LHCI (Fig. 4D) are virtually identical with the corresponding protein conformations in the *C. caldarium* PSI-LHCI (Fig. 4E), with conserved amino acid residues W92/P94 of PsaB, S4/P7/S8/I9/P12/L16 of PsaI, and E72/L76 of PsaL (Fig. 4, D and E). Sequence similarities (identities) for PsaB, PsaI, and PsaL between *G. sulphuraria* and *C. caldarium* are 92% (84%), 75% (53%), and 80% (63%), respectively (fig. S14).

Substantial differences in Chl composition exist around the Q103-T123 loop. The GsPSI-LHCI contains Chl a102 of PsaI (a102/I in Fig. 4D), while the *C. caldarium* PSI-LHCI has Chl a306 of LHCI-1 (a306/1 in Fig. 4E). The *G. sulphuraria* and *C. caldarium* PSI-LHCI structures lack Chl molecules corresponding to the *C. caldarium* a306/1 and the *G. sulphuraria* a102/I, respectively. In the GsPSI-LHCI, a water molecule w201 of PsaI coordinates with the Mg atom of a102/I (Fig. 4, D and F). Despite steric hindrance caused by the two Chls (Fig. 4G), the protein conformation of PsaI, especially residues S8, I9, P12, and L16, remains conserved between the two species (Fig. 4, D to F, and fig. S14). These observations suggest that the absence of RedCAP in the PSI core of *C. caldarium* NIES-2137 may lead to two changes: (i) spatial constraints imposed by PSI proteins around the Q103-T123 loop of RedCAP and/or (ii) loss of a102/I with the addition of a306/1.

We propose that during the evolutionary history of red-lineage algae, RedCAP was present in an ancestral red alga, retained by *G. sulphuraria* and *P. purpureum*, but lost in *C. caldarium* and *C. merolae* (*22*). Considering the characteristics of its PSI-LHCI structure, *G. sulphuraria* appears to share a close relationship with an ancestral red alga, although it belongs to the monophyletic Cyanidiophyceae (*6*, *7*, *19*, *35*).

### Protein-protein interactions of other LHCI subunits

**Fig. 5.**
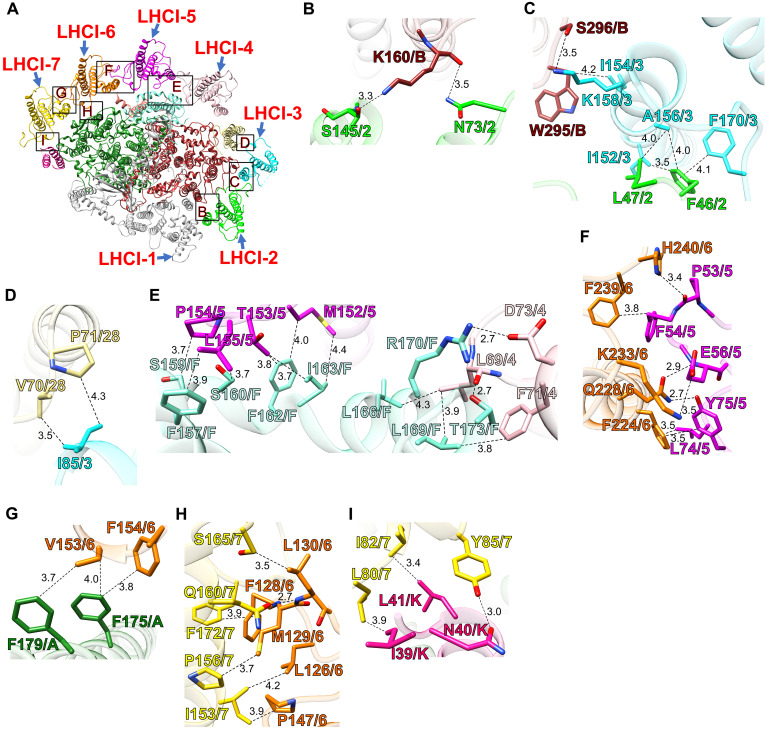
Protein-protein interactions between LHCI and PSI subunits and among LHCIs. (**A**) The PSI-LHCI structure is viewed from the stromal side. The areas encircled by black squares are enlarged in (B) to (I). The PSI and LHCI subunits involved in protein-protein interactions are colored differently. (**B** to **I**) Characteristic amino acid residues involved in protein-protein interactions. Interactions are indicated by dashed lines, and the numbers are distances in angstrom. Amino acid residues participating in the interactions are labeled; for example, K160/B and N73/2 indicate Lys^160^ of PsaB and Asn^73^ of LHCI-2, respectively. A, PsaA; B, PsaB; F, PsaF; K, PsaK; 28, Psa28; 2, LHCI-2; 3, LHCI-3; 4, LHCI-4; 5, LHCI-5; 6, LHCI-6; 7, LHCI-7.

LHCI-2 (Lhcr6) is positioned near PsaB through protein-protein interactions at distances of 3.3 to 3.5 Å at the stromal side, where residues N73/S145 of LHCI-2 interact with K160 of PsaB (Fig. 5B). While LHCI-2 has an Asn at position 73, the other LHCIs have Asp at the corresponding position (fig. S15). In addition, LHCI-2 interacts with LHCI-3 through protein-protein interactions at distances of 3.5 to 4.1 Å at the stromal side between F46/L47 of LHCI-2 and I152/A156/F170 of LHCI-3 (Fig. 5C). Notably, F46 is conserved across the six LHCIs, whereas L47 of LHCI-2 is replaced by Leu, Met, or Phe in the other LHCIs (fig. S15). These results suggest that N73 of LHCI-2 plays a key role in recognizing and preferentially binding to PsaB over LHCI-3.

LHCI-3 (Lhcr3) is positioned near PsaB and Psa28 through interactions between residues I154/K158 of LHCI-3 and W295/S296 of PsaB and between I85 of LHCI-3 and V70/P71 of Psa28, with distances of 3.5 to 4.3 Å at the stromal side (Fig. 5, C and D). Unique to LHCI-3, positions 85, 154, and 158 contain Ile, Ile, and Lys, respectively, which are absent in the other LHCIs (fig. S15). This unique combination likely facilitates the specific recognition and preferential binding of I85 and I154/K158 to Psa28 and PsaB, respectively.

LHCI-4 (Lhcr5) is positioned near PsaF, interacting through residues L69/F71/D73 of LHCI-4 with L166/L169/R170/T173 of PsaF at distances of 2.7 to 4.3 Å at the stromal side (Fig. 5E). LHCI-4 uniquely has Phe and Asp at positions 71 and 73 (fig. S15), suggesting that this distinctive combination enables selective recognition of the binding site on PsaF.

LHCI-5 (Lhcr4) is also positioned near PsaF, where residues M152/T153/P154/L155 of LHCI-5 interact with F157/S159/S160/F162/I163 of PsaF at distances of 3.7 to 4.4 Å at the stromal side (Fig. 5E). The Met, Thr, and Pro residues at positions 152, 153, and 154 in LHCI-5 are unique among the six LHCIs (fig. S15), supporting its preferential binding to PsaF. Furthermore, LHCI-5 interacts with LHCI-6 through protein-protein interactions between P53/F54/E56/L74/Y75 of LHCI-5 and F224/Q228/K233/F239 of LHCI-6 at distances of 2.7 to 3.8 Å at the stromal side (Fig. 5F). However, these residues are not unique to LHCI-5 (fig. S15), suggesting that M152/T153/P154 of LHCI-5 are critical for its preferential binding to PsaF rather than LHCI-6.

LHCI-6 (Lhcr1) is positioned near PsaA through interactions between residues V153/F154 of LHCI-6 and F175/F179 of PsaA at distances of 3.7 to 4.0 Å at the stromal side (Fig. 5G). LHCI-6 contains a unique combination of Val and Phe at these positions (fig. S15), facilitating specific binding to PsaA. LHCI-6 also interacts with LHCI-7 via residues L126/F128/M129/L130/P147 of LHCI-6 and I153/P156/Q160/S165/F172 of LHCI-7 at distances of 3.5 to 4.2 Å at the stromal side (Fig. 5H). LHCI-6 has Met, Leu, Phe, and Lys at positions 129, 130, 224, and 233, respectively, a unique combination among the six LHCIs (fig. S15). This distinct set of residues (M129/L130/F224/K233) likely plays an important role in the recognition and preferential binding of LHCI-6 to PsaA, LHCI-5, and LHCI-7.

LHCI-7 (Lhcr2) is positioned near PsaK, interacting through residues L80/I82/Y85 of LHCI-7 with I39/N40/L41 of PsaK at distances of 3.0 to 3.9 Å at the stromal side (Fig. 5I). LHCI-7 contains Tyr, Pro, and Ser at positions 85, 156, and 165, respectively, which are unique among the six LHCIs (fig. S15), suggesting that Y85/P156/S165 of LHCI-7 are crucial for recognizing and preferentially binding to PsaK and LHCI-6.

## DISCUSSION

Kumazawa and Ifuku (*25*) have reported a comprehensive phylogenetic analysis of red algal LHCs, classifying red algal Lhcrs into groups I to VII and a Galdieriales-specific Lhcr clade. Among the six Lhcrs of *G. sulphuraria* (GsLhcr1 to GsLhcr6), GsLhcr6, GsLhcr5, GsLhcr4, GsLhcr1, and GsLhcr2 were categorized into groups I, IV, V, VI, and VII, respectively, and corresponded to their orthologs in *P. purpureum*, namely, PpLhcr2 (group I), PpLhcr1 (group IV), PpLhcr5 (group V), PpLhcr4 (group VI), and PpLhcr3 (group VII). In addition, GsLhcr4, GsLhcr1, and GsLhcr2 were identified as the orthologs of *C. merolae* Lhcr1 (CmLhcr1), CmLhcr2, and CmLhcr3, respectively, while GsLhcr3 was uniquely classified into the Galdieriales-specific clade, with no orthologs in *P. purpureum* or *C. merolae*.

**Fig. 6.**
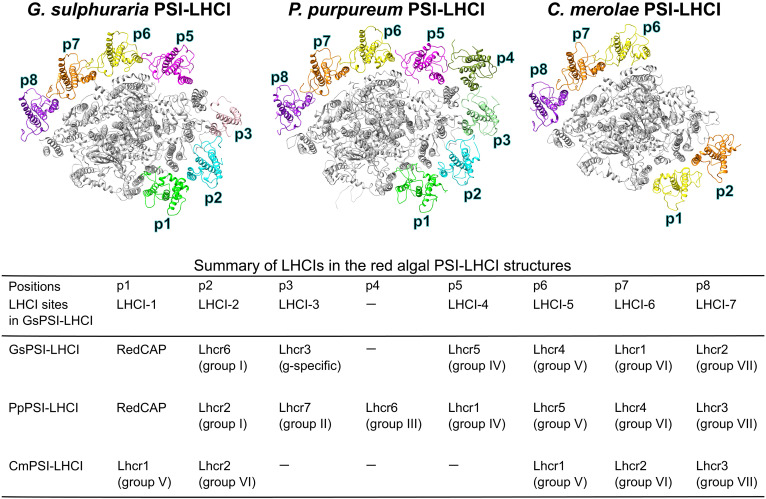
Structural and phylogenetic diversity of red algal LHCIs. The PSI-LHCI structures of the three red algae, *G. sulphuraria* (this study), *P. purpureum* (PDB: 7Y5E), and *C. merolae* (PDB: 5ZGB), are viewed from the stromal side. Each LHCI position is labeled p1 to p8 based on the *P. purpureum* PSI-LHCI. PSI subunits are shown in gray, whereas LHCI subunits are depicted in various other colors. LHCI subunits belonging to the same group are shown in the same color. The table summarizes the correlations between binding positions (p1 to p8) and each of gene products identified, LHCI sites in the GsPSI-LHCI, and groups defined previously (*25*).

On the basis of the phylogenetic analysis (*25*), structural comparisons and orthologous relationships among red algal LHCIs are summarized in Fig. 6. According to Kumazawa and Ifuku (*25*), the PSI-LHCI structures of *C. merolae*, *P. purpureum*, and *G. sulphuraria* are used, with *C*. *caldarium* RK-1 (NIES-2137) exhibiting structural consistency with *C. merolae* (*22*). Binding positions are assigned as p1 to p8 according to the PSI-LHCI of *P. purpureum* (PpPSI-LHCI), which has the largest number of LHCI subunits among the three species. In GsPSI-LHCI, the binding sites of LHCI subunits correspond to LHCI-1 (p1), LHCI-2 (p2), LHCI-3 (p3), LHCI-4 (p5), LHCI-5 (p6), LHCI-6 (p7), and LHCI-7 (p8).

The GsLhcrs at the LHCI-2 and LHCI-4 to LHCI-7 sites in GsPSI-LHCI correspond to their orthologs at the respective sites of PpPSI-LHCI, whereas GsLhcr3 (a Galdieriales-specific Lhcr clade) at the LHCI-3 site in GsPSI-LHCI aligns with PpLhcr7 (group II) at the corresponding site in PpPSI-LHCI, despite their lack of orthology. In contrast, the GsLhcrs at the LHCI-5 to LHCI-7 sites in GsPSI-LHCI correspond to orthologs of the PSI-LHCI of *C. merolae* (CmPSI-LHCI) at the respective sites, although GsLhcr6 at the LHCI-2 site in GsPSI-LHCI is replaced by CmLhcr2 (group VI) at the corresponding site in CmPSI-LHCI. These observations indicate that the group VI Lhcr at the LHCI-2 site in CmPSI-LHCI has been acquired secondarily after the divergence of Galdieriales from other Cyanidiophyceae including Cyanidiales and Cyanidioschyzonales, supporting neolocalization of this LHCI based on combined structural and phylogenetic analysis (*22*).

We compared amino acid residues of LHCIs among the three red algal PSI-LHCI structures (fig. S16) based on the protein-protein interaction data from GsPSI-LHCI (Figs. 4 and 5) and the summary of structural comparisons and orthologous relationships among LHCI subunits (Fig. 6). The amino acid residues involved in protein-protein interactions between LHCI and PSI subunits are largely conserved between *G. sulphuraria* and *C. merolae*/*P. purpureum* at the LHCI-4, LHCI-6, and LHCI-7 sites (fig. S16, B, D, and E), whereas they differ at the LHCI-2 and LHCI-5 sites (fig. S16, A and C).

It is of particular interest that the binding properties of fucoxanthin Chl *a*/*c*–binding proteins (FCPs) in the PSI-FCPI structures between *C*. *gracilis* and *T*. *pseudonana* are highly conserved (*34*). The orthologous relationship and amino acid residues involved in interactions closely match between the two diatoms. In contrast, among the three red algae, the conservation of amino acid residues involved in protein-protein interactions is significantly low, despite clear orthologous relationship among LHCIs. These observations imply that protein-protein interactions between LHCI and PSI subunits were less conserved during the early evolutionary stages of red algae but became highly conserved following secondary endosymbiosis, as seen in diatoms.

**Fig. 7.**
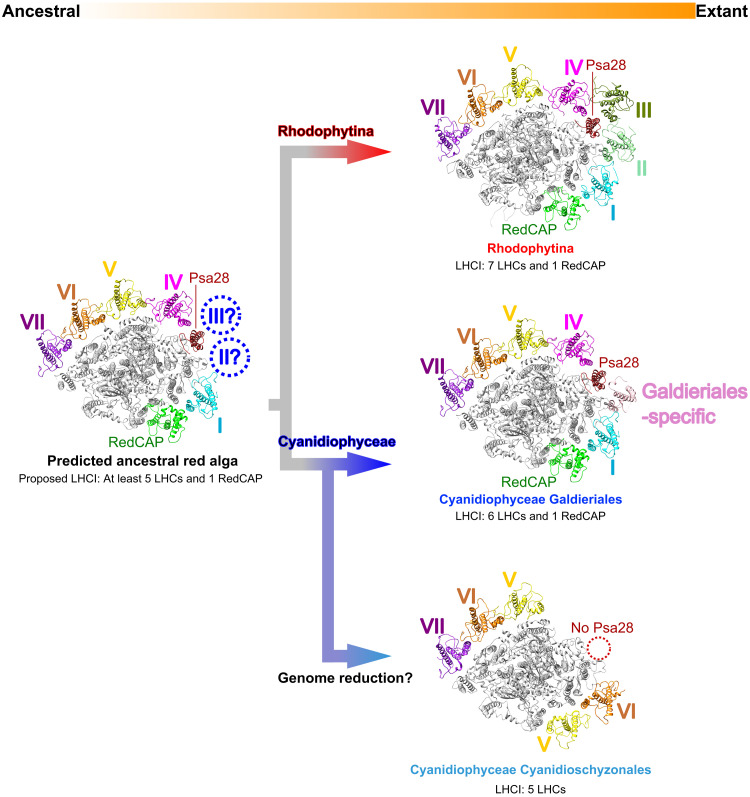
Evolutionary scheme of LHCI subunits in red algal PSI-LHCI supercomplexes. The PSI-LHCI structures are viewed from the stromal side. LHCI and Psa28 subunits are colored, while other PSI subunits are shown in gray. LHCI subunits belonging to the same group are shown in the same color. I to VII, groups I to VII of Lhcrs. An ancestral red algal PSI-LHCI structure is provisionally depicted using the GsPSI-LHCI structure, whereas the other PSI-LHCI structures originate from the Rhodophytina *P. purpureum* (PDB: 7Y5E), the Galdieriales *G. sulphuraria* (this study), and the Cyanidioschyzonales *C. merolae* (PDB: 5ZGB).

This study has markedly advanced our understanding of the evolutionary trajectory of PSI-LHCI in red algae by elucidating the structure of PSI-LHCI from *G. sulphuraria*, a member of the Galdieriales—the earliest diverging group within Cyanidiophyceae. Leveraging these findings, we propose a model for an ancestral red algal PSI-LHCI supercomplex (Fig. 7), which likely features RedCAP and Lhcrs from groups I and IV to VII in conserved positions across both Rhodophytina and Galdieriales, although these components are absent in Cyanidioschyzonales. It is noteworthy that LHCIs in Cyanidioschyzonales lack ancestral traits at positions 1 and 2 due to neolocalization, reflecting LHCI reorganization during genome reduction (*22*). In addition, we hypothesize that Psa28 was present in an ancestral red algal PSI-LHCI, fulfilling a pivotal role in group IV Lhcr binding.

The evolutionary trajectory of LHCI subunits at positions 3 and 4, as shown in Fig. 6, permits us to propose plausible scenarios (fig. S17). For position 3, it remains conceivable that either the group II Lhcr in PpPSI-LHCI or the Galdieriales-specific Lhcr in GsPSI-LHCI originated from a common ancestor. Alternatively, both may have arisen independently after divergence from a common ancestor. The close interactions between group II and group III Lhcrs within Rhodophytina PSI-LHCI suggest that these Lhcrs were acquired concurrently, irrespective of the exact timing of acquisition. This results in six equally plausible evolutionary scenarios based on the current Lhcr phylogenetic topology. Consequently, reconstructing the complete architecture of an ancestral red algal PSI-LHCI remains a challenge. Future research integrating both structural and phylogenetic analyses of red-lineage LHCs may offer critical insights into the binding mechanisms of LHCI subunits, including the identification of secondarily acquired Lhcrs. This approach could potentially aid in reconstructing the ancestral PSI-LHCI structure.

## MATERIALS AND METHODS

### HPLC analysis

Culture of the *G. sulphuraria* NIES-3638 cells from the Microbial Culture Collection at National Institute for Environmental Studies (NIES), Japan, and preparation of the PSI-LHCI supercomplex were performed, as described previously (*27*). Quinone molecules were analyzed using a Shimadzu HPLC system (*46*) equipped with a reversed-phase InertSustain C18 column (250 mm by 4.6 mm, 5-μm particle size; GL Sciences). Quinone and pigment molecules in the prepared *G. sulphuraria* PSI cores (*27*) were extracted using 100% methanol. The samples were subjected to HPLC and eluted with solvent A (100% methanol) and solvent B (100% isopropanol) in a linear gradient: 0 to 10 min, 0% solvent B; 10 to 30 min, 0 to 97% solvent B; and 30 to 40 min, 97% solvent B. The flow rate was set to 0.5 ml min^−1^, with detection at 270 nm. Phylloquinone (P0642, Tokyo Chemical Industry) and ubiquinone-4 (C2470, Sigma) were purchased as standards.

### Cryo-EM data collection

A 3-μl aliquot of the GsPSI-LHCI supercomplex (2.5 mg of Chl ml^−1^) in 20 mM Mes-NaOH (pH 6.5) buffer containing 0.5 M betaine, 5 mM CaCl_2_, 10 mM MgCl_2_, and 0.03% *n*-dodecyl-β-d-maltoside was applied to a Quantifoil R1.2/1.3 Cu 300 mesh grid in the chamber of FEI Vitrobot Mark IV (Thermo Fisher Scientific). Then, the grid was blotted with a filter paper for 4 s at 4°C under 100% humidity and plunged into liquid ethane cooled by liquid nitrogen. The frozen grid was transferred into a CRYO ARM 300 electron microscope (JEOL) equipped with a cold-field emission gun operated at 300 kV. All image stacks were collected from 5 × 5 holes per stage adjustment to the central hole, and image shifts were applied to the surrounding holes while maintaining an axial coma-free condition. The images were recorded with an in-column energy filter with a slit width of 20 eV and at a nominal magnification of ×60,000 on a direct electron detector (Gatan K3, AMETEK). The nominal defocus range was −1.8 to −1.2 μm. Physical pixel size corresponded to 0.752 Å. Each image stack was exposed at a dose rate of 25.34 e^−^ Å^−2^ s^−1^ for 1.97 s in correlated double sampling (CDS) mode, with dose-fractionated 50 movie frames. In total, 8800 image stacks were collected.

### Cryo-EM image processing

The resultant movie frames were aligned and summed using MotionCor2 (*47*) to yield dose-weighted images. Estimation of the contrast transfer function was performed using CTFFIND4 (*48*). All of the following processes were performed using RELION-4.0 (*49*). In total, 2,583,694 particles were automatically picked up and used for reference-free two-dimensional (2D) classification. Then, 933,316 particles were selected from good 2D classes and subsequently subjected to 3D classification without any symmetry. An initial model for the first 3D classification was generated de novo from 2D classification. As shown in fig. S1C, the final PSI-LHCI structure was reconstructed from 110,313 particles. The overall resolution of the cryo-EM map was estimated to be 2.19 Å by the gold standard Fourier Shell Correlation (FSC) curve with a cutoff value of 0.143 (fig. S2A) (*50*). Local resolutions were calculated using RELION (fig. S2C).

### Model building and refinement

Two types of the cryo-EM maps were used for the model building of the PSI-LHCI supercomplex: One was a postprocessed map, and the other was a denoised map using Topaz version 0.2.4 (*51*). The postprocessed map was denoised using the trained model in 100 epochs with two half-maps. Homology models of each subunit in the PSI-LHCI supercomplex were built using the Phyre2 server (*52*), and then their structures were inspected and manually adjusted against the maps with Coot (*53*). Each model was built on the basis of interpretable features from the density maps at a contour level of 1.5 σ in the denoised and postprocessed maps. For the assignment of Cars, BCR, BCX, and ZXT were distinguished on the basis of the density covering the head group of Cars with the above thresholds. The PSI-LHCI structure was refined with phenix.real_space_refine (*54*) and Servalcat (*55*), with geometric restraints for the protein-cofactor coordination. The final model was validated with MolProbity (*56*), EMRinger (*57*), and *Q*-score (*58*). The statistics for all data collection and structure refinement are summarized in tables S1 and S2. All structural figures were made by PyMOL (*59*), UCSF Chimera (*60*), and UCSF ChimeraX (*61*).

Because the numbering of Chls, Cars, and other cofactors in this paper was different from those of the Protein Data Bank (PDB) data, we listed the relationship of their numbering in this paper with those in the PDB data in tables S6 to S8.

### Phylogenetic analysis

Psa28 sequences were collected from National Center for Biotechnology Information (NCBI) nr, NCBI tsa-nr, Joint Genome Institute (JGI) Phycocosm, and EukProt v3 (https://evocellbio.com/eukprot/) (*62*), and nuclear draft genome assemblies were deposited in a public repository using BLASTP (*63*). Accession numbers were provided on the molecular phylogenetic tree of Psa28. The Psa28 sequences of *Diacronema viridis*, *Phaeocystis jahnii*, and *Phaeocystis rex* are derived from their draft genomes (*63*). Amino acid sequences of Psa28 were manually curated and aligned using MAFFT E-INS-i v7.525 (*64*). The alignment was trimmed using ClipKit v2.2.0 with smart-gap mode (*64*). The phylogenetic tree was inferred using IQ-TREE 2.2.2.7 (*65*) with the Q.pfam+F+R5 model (*66*) selected by ModelFinder (*67*). The tree was visualized by iTOL v6 (*68*) and rooted with the Galdieriales clade. Ultrafast bootstrap approximation was performed with 1000 replicates (*66*). SH-like approximate likelihood ratio test (SH-aLRT) support (%) tests with 1000 replicates and aBayes support tests were also performed (*69*, *70*).
