# Supplementary material for: Structure of a photosystem I supercomplex from Galdieria sulphuraria close to an ancestral red alga
Source: Sci Adv. 2025 May 16;11(20):eadv7488. doi: 10.1126/sciadv.adv7488 (PMC12083527; doi:10.1126/sciadv.adv7488)
Supplement: Supplementary file 1 — Figs. S1 to S17 Tables S1 to S8 [file sciadv.adv7488_sm.pdf]

Science Advances
AAAS

Supplementary Materials for

Structure of a photosystem I supercomplex from *Galdieria sulphuraria* close to an ancestral red alga

Koji Kato *et al.*

Corresponding author: Jian-Ren Shen, shen@cc.okayama-u.ac.jp; Kentaro Ifuku, ifuku.kentaro.2m@kyoto-u.ac.jp; Ryo Nagao, nagry@shizuoka.ac.jp

Sci. Adv. **11**, eadv7488 (2025)
DOI: 10.1126/sciadv.adv7488

This PDF file includes:

Figs. S1 to S17
Tables S1 to S8

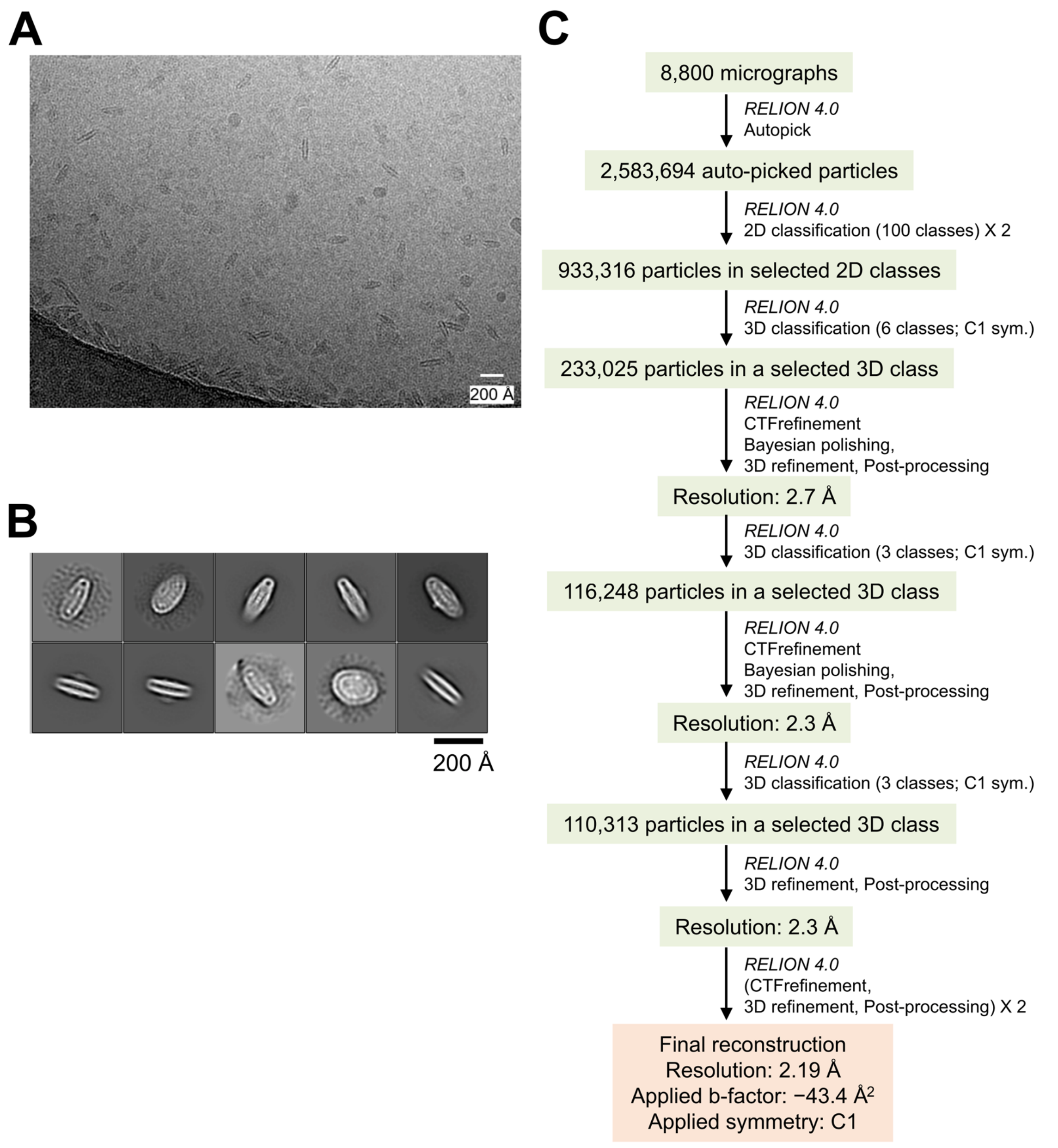

Fig. S1 | Cryo-EM data collection and processing of PSI-LHCI.

A, A representative cryo-EM micrograph of PSI-LHCI from 8,800 micrographs. **B,** Representative 2D classes of PSI-LHCI. The box size is 361 Å. **C,** A schematic flowchart showing the classification scheme and data processing for PSI-LHCI. The overall PSI-LHCI structure was reconstructed at a resolution of 2.19 Å from 110,313 particles. See Methods section for more details.

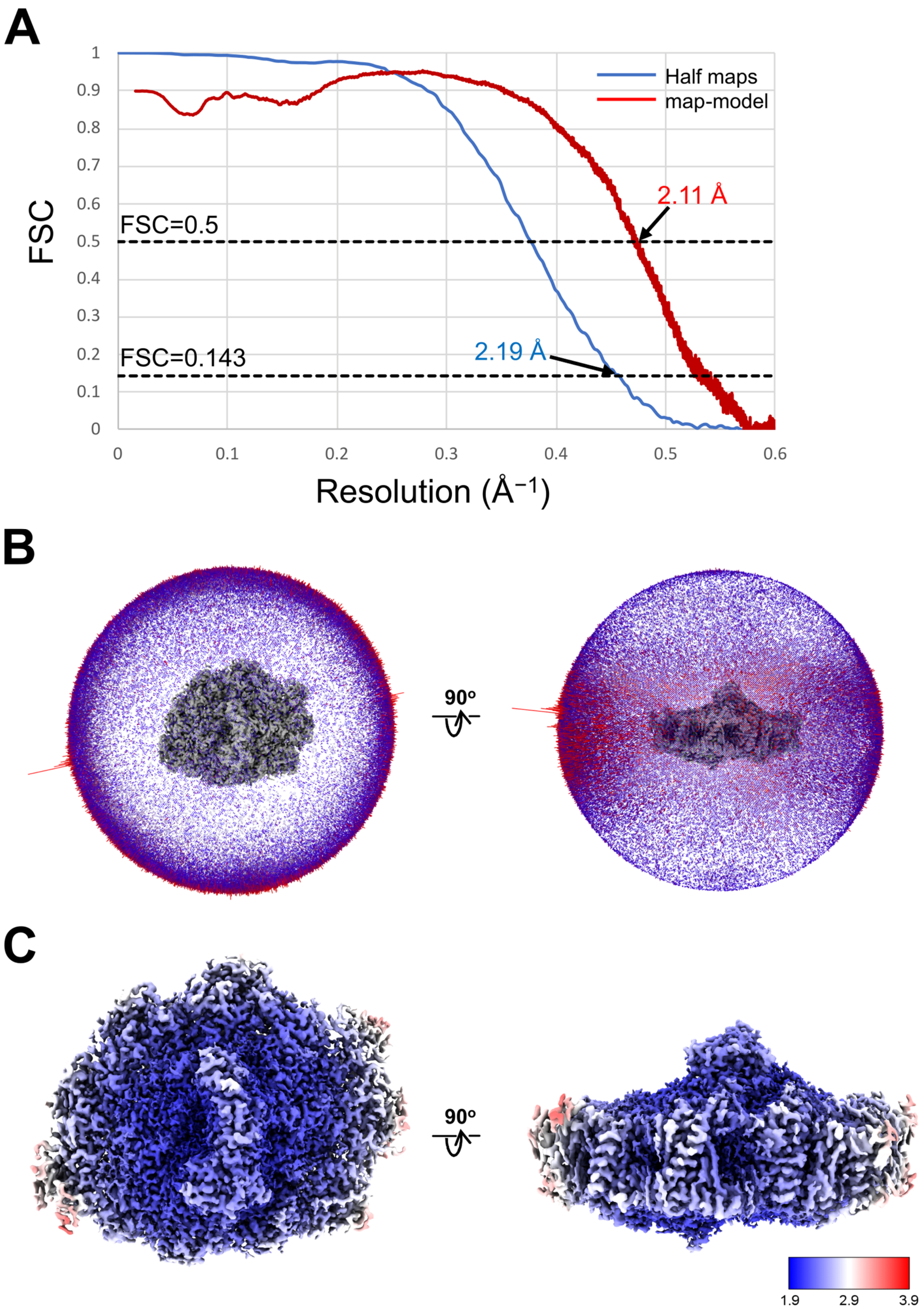

Fig. S2 | Evaluation of the cryo-EM map quality.

A, FSC curves of PSI-LHCI for independently refined half maps (blue) and map-minus-model (red). **B,** Angular distributions of the particles used for the reconstruction of PSI-LHCI. Each cylinder represents one view, and the height of the cylinder is proportional to the number of particles for that view. **C,** Local resolution maps of PSI-LHCI.

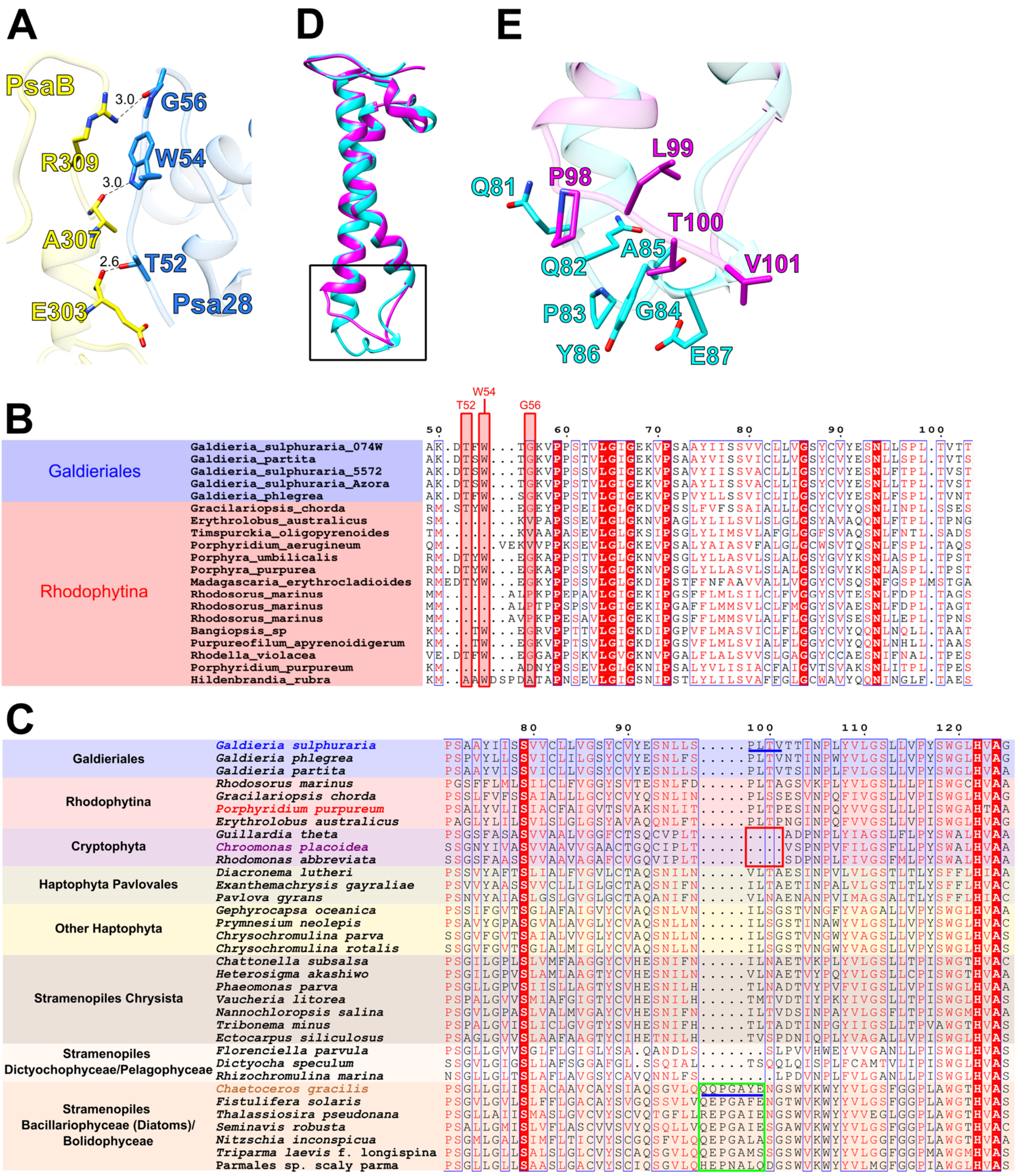

Fig. S3 | Structural and evolutionary characteristics of Psa28.

A, Protein-protein interactions between Psa28 and PsaB in the *G. sulphuraria* PSI-LHCI structure. Interactions are indicated by dashed lines, and the numbers are distances in Å. **B,** Alignment of red algal Psa28 around the region interacting with PsaB indicated in panel **A**. The residues involved in the interactions (T52, W54, and G56 in *G. sulphuraria* Psa28) are indicated as red open boxes. **C,** Alignment of Psa28 around its lumenal region. The motifs characteristic of each taxon are indicated with boxes. **D,** Structural comparison of Psa28 between *G. sulphuraria* (magenta) and *C. gracilis* (cyan, PDB: 6L4U). The area encircled by a black square is enlarged in panel **E**. **E,** An expanded view of the Psa28 loop on the lumenal side. The amino acid residues displayed and labeled in magenta correspond to the sequences highlighted by blue underlines in panel **C**.

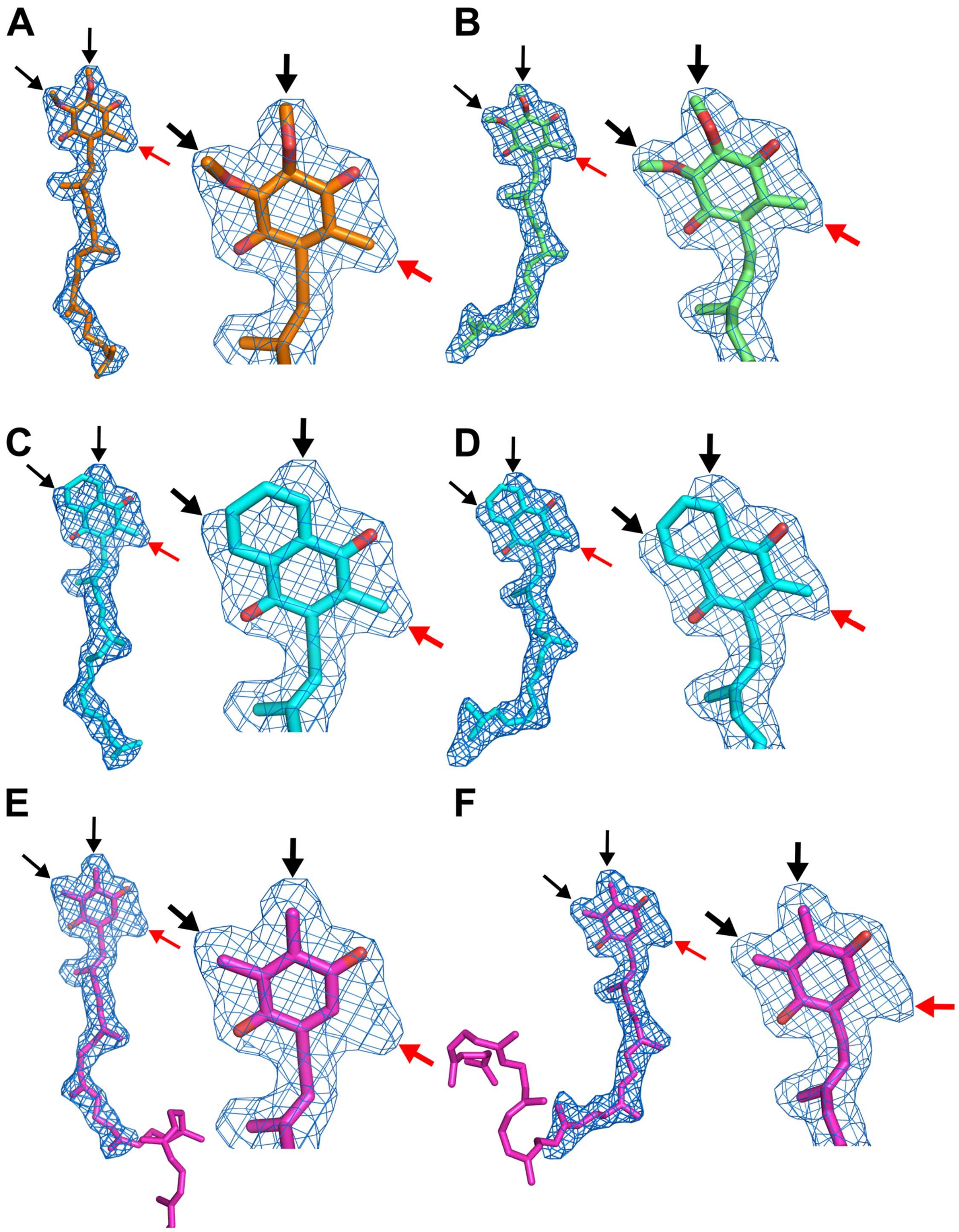

Fig. S4 | Evaluation of the structures of A_1.

Cryo-EM maps (3 σ) and models of the A_1 molecules in PsaA (**A, C, E**) and PsaB (**B, D, F**). Ubiquinone-4 (**A, B**); phylloquinone (**C, D**); plastoquinone-9 (**E, F**). Each panel displays the overall structure (left) and an expanded view of the ring structure (right). Characteristic map features are indicated by black and red arrows (see text).

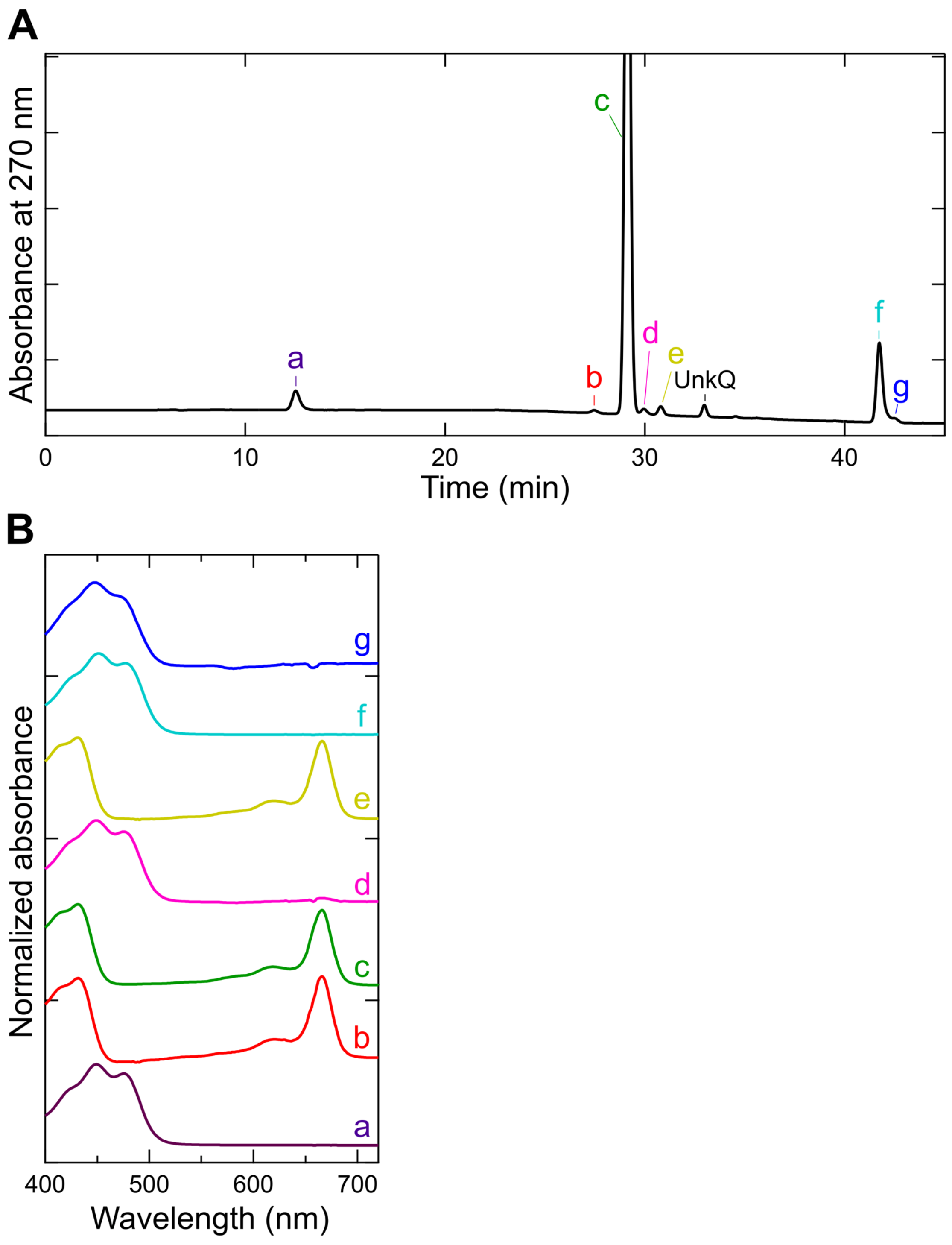

Fig. S5 | Identification of pigment molecules.

(**A**) Detectable peaks are labeled a–g. The HPLC chromatogram corresponds to the black line in Fig. 3C. UnkQ denotes an unknown quinone. (**B**) Absorption spectra of peaks a–g. The spectra were measured by a photodiode array detector in the HPLC system and normalized by the maximum peak intensity of each spectrum.

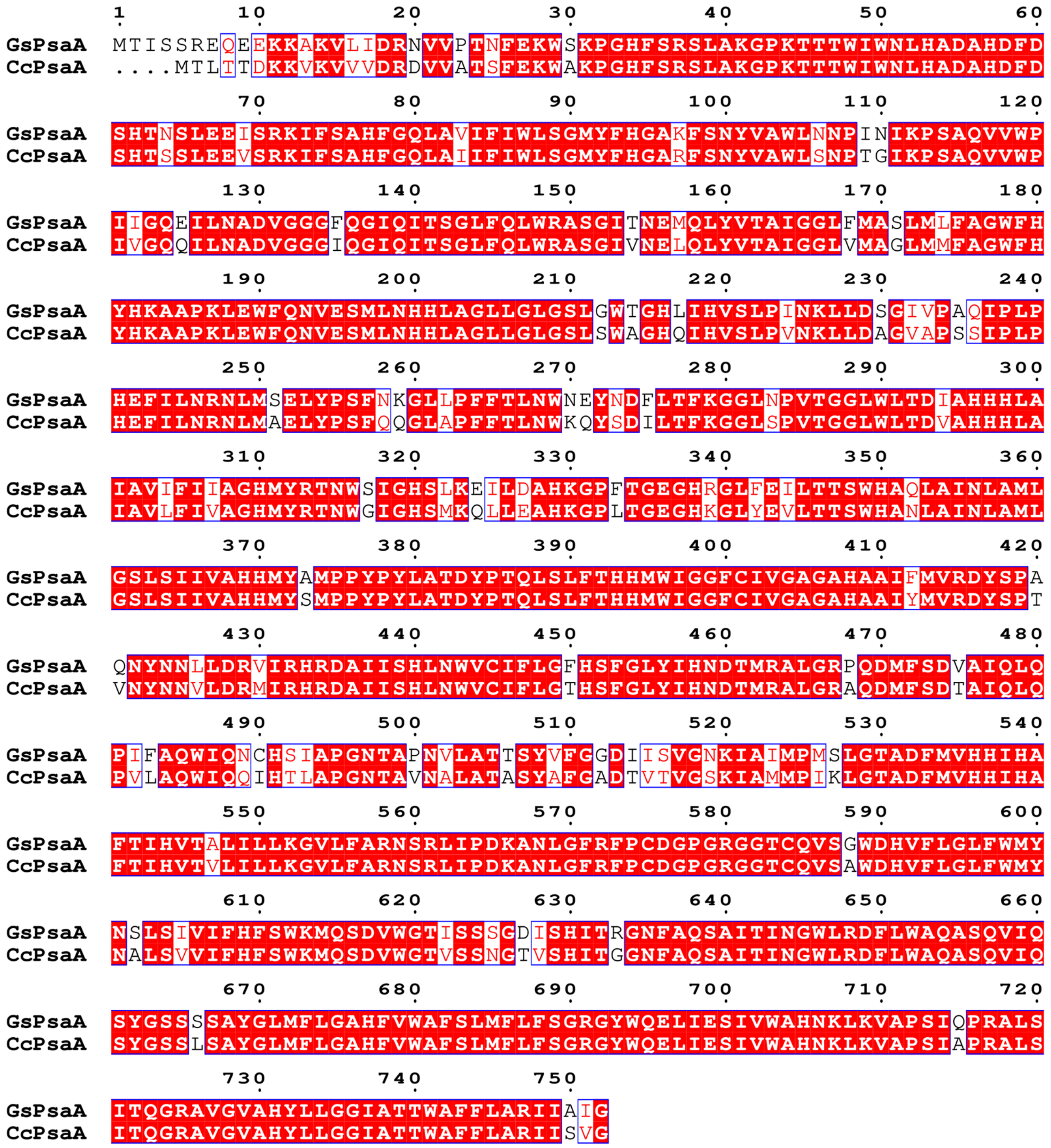

Fig. S6 | Sequence alignment of PsaA between *G. sulphuraria* and *C. caldarium*.

Sequence alignment of PsaA proteins was performed using ClustalW (https://www.genome.jp/tools-bin/clustalw) and ESPript (https://espript.ibcp.fr/ESPript/cgi-bin/ESPript.cgi). GsPsaA, PsaA of *G. sulphuraria* NIES-3638; CcPsaA, PsaA of *C. caldarium* NIES-2137.

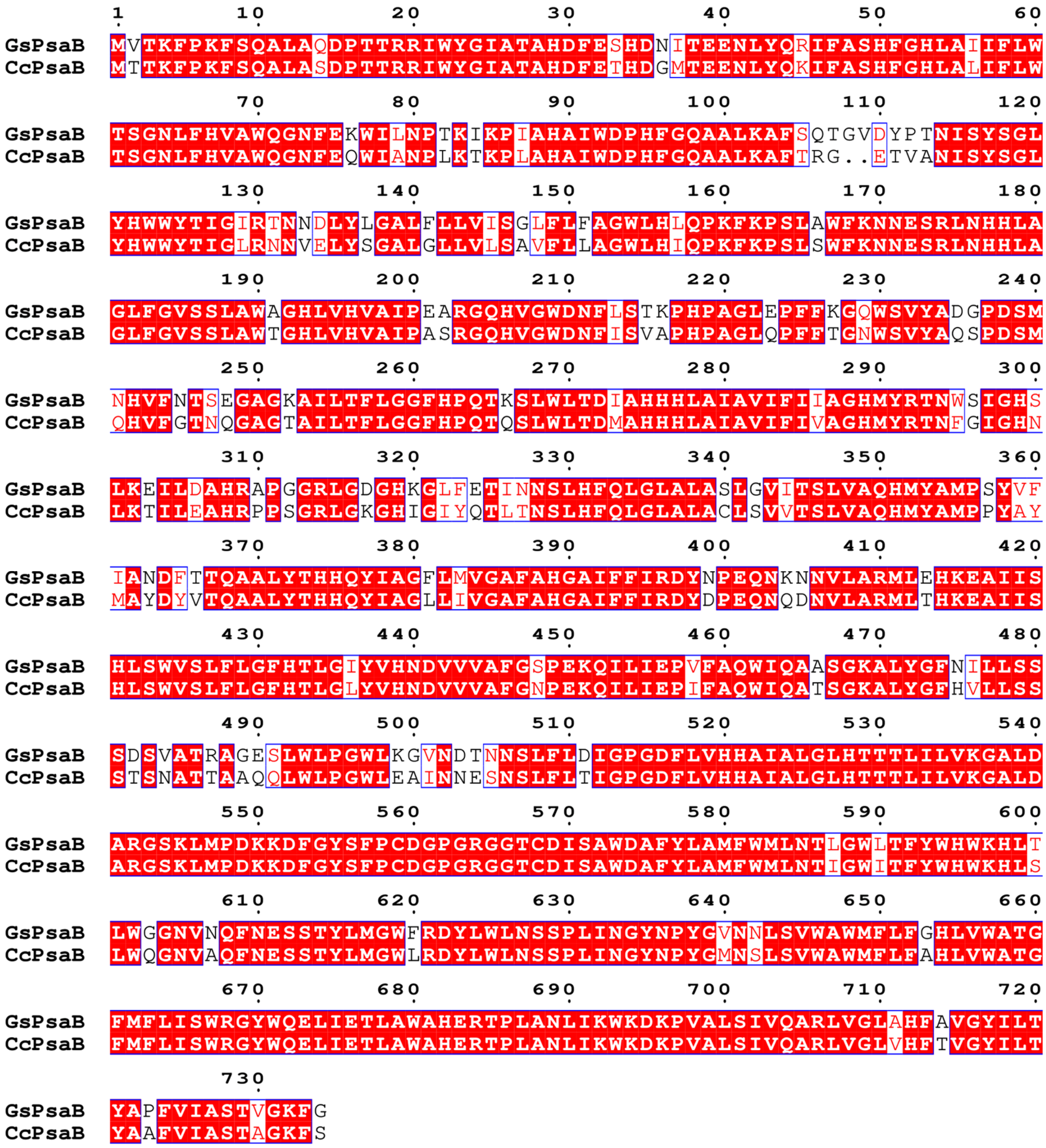

Fig. S7 | Sequence alignment of PsaB between *G. sulphuraria* and *C. caldarium*.

Sequence alignment of PsaB proteins was performed using ClustalW and ESPript. GsPsaB, PsaB of *G. sulphuraria* NIES-3638; CcPsaB, PsaB of *C. caldarium* NIES-2137.

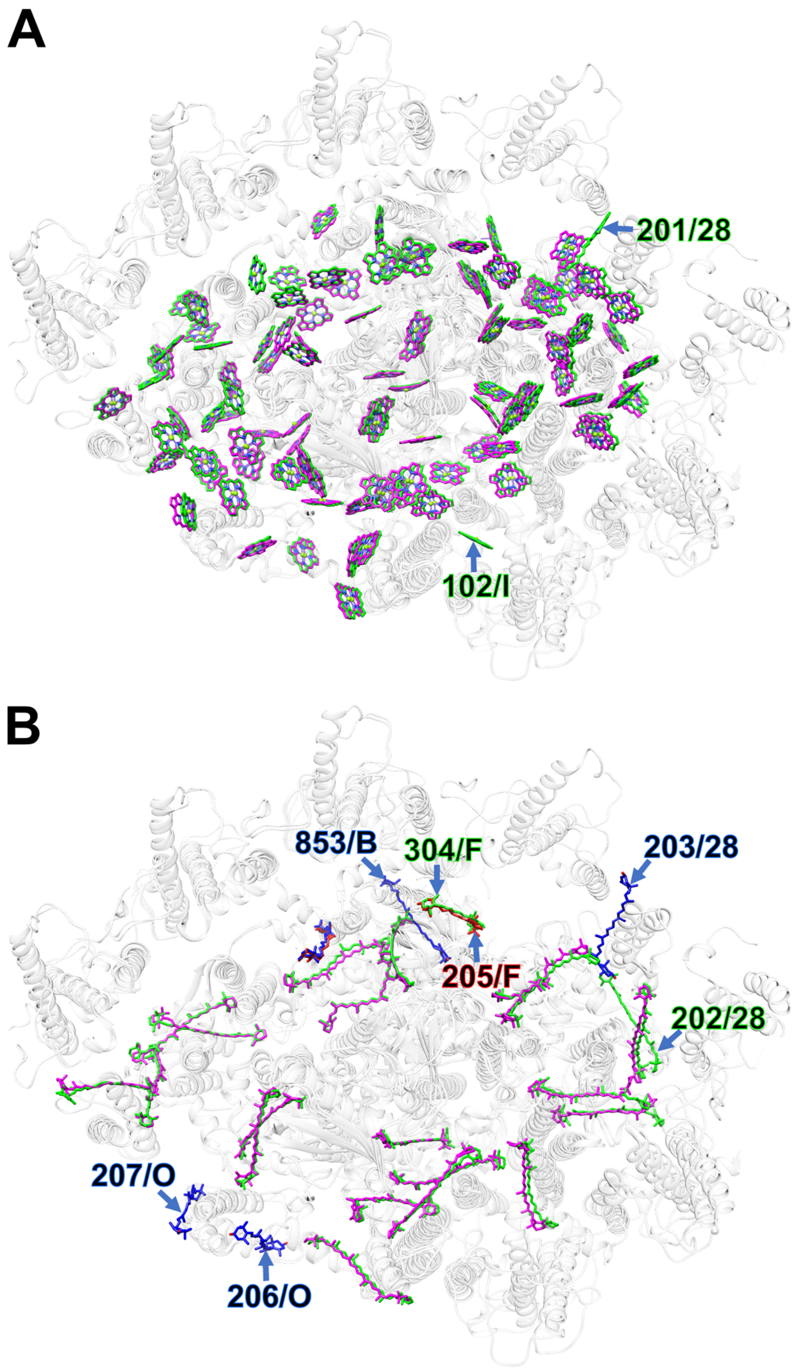

Fig. S8 | Comparison of Chls and Cars of the PSI cores between *G. sulphuraria* and *C. caldarium*.

The *G. sulphuraria* PSI-LHCI structure was overlapped with the *C. caldarium* PSI-LHCI structure (PDB: 8WEY) viewed from the stromal side. Chls and Cars are shown as sticks, and proteins are shown as cartoons. Only rings of the Chl molecules are depicted. **A,** Chls of the PSI cores of *G. sulphuraria* and *C. caldarium* colored green and magenta, respectively. Unique Chls are labeled; 201/28 and 102/I indicate Chl201 of Psa28 and Chl102 of PsaI, respectively. **B,** Cars of the PSI cores of *G. sulphuraria* and *C. caldarium*. Green, BCRs of *G. sulphuraria*; magenta, BCRs of *C. caldarium*; blue, ZXTs of *G. sulphuraria*; red, ZXTs of *C. caldarium*. Unique Cars are labeled; for example, 203/28 means ZXT202 of Psa28. B, PsaB; F, PsaF; J, PsaJ; O, PsaO.

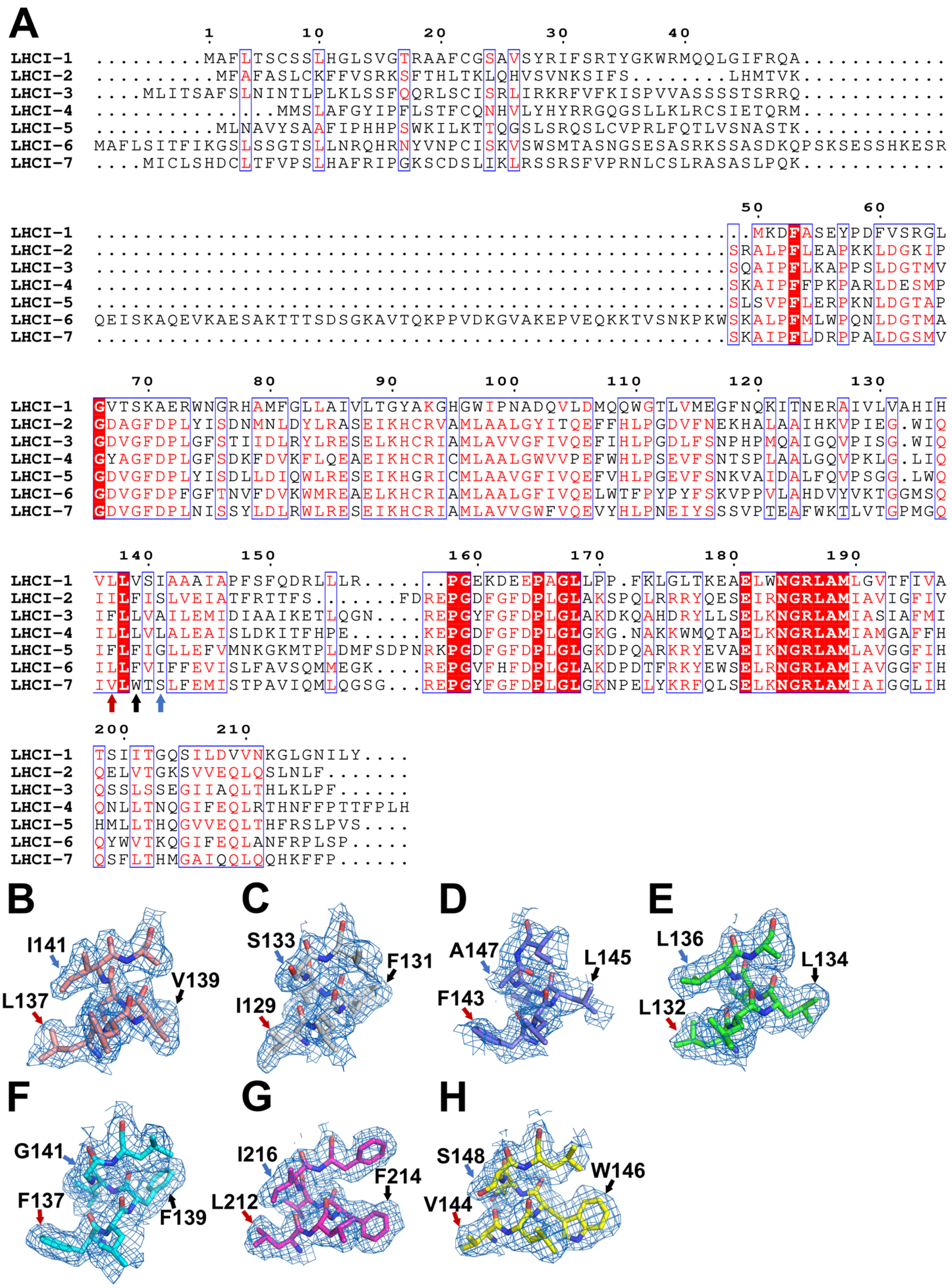
A
1 10 20 30 40
LHCI-1 .........MAFLTSCSSLHGLSVGTRAAFCGSAVSYRIFSRTYGKWRMQQLGIFRQA............
LHCI-2 ..........MFAFASLCKFFVSRKSFTHLTKLQHVSVNKSIFS........LHMTVK............
LHCI-3 ....MLITSAFSLNINTLPLKLSSFQQRLSCISRLIRKRFVFKISPVVASSSSTSRRQ............
LHCI-4 .............MMSLAFGYIPFLSTFCQNHVLYHYRRGQGSLLKLRCSIETQRM............
LHCI-5 ..........MLNAVYSAAFIPHHPSWKILKTTQGSLSRQSLCVPRLFQTLVSNASTK............
LHCI-6 MAFLSITFIKGSLSSGTSLLNRQHRNYVNPCISKVSWSMTASNGSESASRKSSASDKQPSKSESSHKESR
LHCI-7 ....MICLSHDCLTFVPSLHAFRIPGKSCDSLIKLRSSRSFVPRNLCSLRASASLPQK............
50 60
LHCI-1 ....MKDFASEYPDFVSRGL
LHCI-2 ....SRALPFLEAPKKLDGKIP
LHCI-3 ....SQAIPFLKAPPSLDGTMV
LHCI-4 ....SKAIPFFPKPARLDESMP
LHCI-5 ....SLSVPFLERPKNLDGTAP
LHCI-6 QEISKAQEVKAESAKTTTSDSGKAVTQKPPVDKGVAKEPVEQKKTVSNKPKWSKALPFMLWPQNLDGTMA
LHCI-7 ....SKAIPFLDRPPALDGSMV
70 80 90 100 110 120 130
LHCI-1 GVTSKAERWNGRHAMFGLLAIVLTGYAKGHGWIPNADQVLDMQQWGTLVMEGFNQKITNERAIVLVAHIH
LHCI-2 GDAGFDPLYISDNMNLDYLRASEIKHCRVAMLAALGYITQEFFHLPGDVFNEKHALAAIHKVPIEG.WIQ
LHCI-3 GDVGFDPLGFSTIIDLRYLRESELKHCRIAMLAVVGFIVQEFIHLPGDLFSNPHPMQAIGQVPISG.WIQ
LHCI-4 GYAGFDPLGFSDKFDVKFLQEAEIKHCRICMLAALGWVVPEFWHLPSEVFSNTSPLAALGQVPKLG.LIQ
LHCI-5 GDVGFDPLYISDLLDIQWLRESEIKHGRICMLAAVGFIVQEFVHLPGEVFSNKVAIDALFQVPSGG.LWQ
LHCI-6 GDVGFDPFGFTNVFDVKWMREAELKHCRIAMLAALGFIVQELWTFPYPYFSKVPPVLAHDVYVKTGGMSQ
LHCI-7 GDVGFDPLNISSYLDLRWLRESEIKHCRIAMLAVVGWFVQEVYHLPNEIYSSSVPTEAFWKTLVTGPMGQ
140 150 160 170 180 190
LHCI-1 VLLVSIAAAIAPFSFQDRLLLR........PGEKDEEPAGLLPP.FKLGLTKEAELWNGRLAMLGVTFIVA
LHCI-2 IILFISLVEIATFRTTFS......FDREPGDFGFDPLGLAKSPQLRRYQESEIRNGRLAMIAVIGFIV
LHCI-3 IFLLVAILEMIDIAAIKETLQGN....REPGYFGFDPLGLAKDKQAHDRYLLSELKNGRLAMIASIAFMI
LHCI-4 ILLVLALEAISLDKITFHPE......KEPGDFGFDPLGLGKG.NAKKWMQTAELKNGRLAMIAMGAFFH
LHCI-5 IFLFIGLLEFVMNKGKMTPLDMFSDPNRKPGDFGFDPLGLGKDPQARKRYEVAEIKNGRLAMLAVGGFIH
LHCI-6 ILLFVIFFEVISLFAVSQMMEGK....REPGVFHFDPLGLAKDPDTFRKYEWSELRNGRLAMIAVGGFIH
LHCI-7 IVLWTSLFEMISTPAVIQMLQGSG...REPGYFGFDPLGLGKNPELYKRFQLSELKNGRLAMIAIGGLIH
200 210
LHCI-1 TSIITGQSILDVVNKGLGNILY....
LHCI-2 QELVTGKSVVEQLQSLNLF.......
LHCI-3 QSSLSSEGIIAQLTHLKLPF......
LHCI-4 QNLLTNQGIFEQLRTHNFPTTFPLH
LHCI-5 HMLLTHQGVVEQLTHFRSLPVS....
LHCI-6 QYWVTKQGIFEQLANFRPLPSP....
LHCI-7 QSFLTHMGAIQQLQQHKFFP......
B
I141
V139
L137
C
S133
F131
I129
D
A147
L145
F143
E
L136
L134
L132
F
G141
F137
F139
G
I216
F214
L212
H
S148
W146
V144

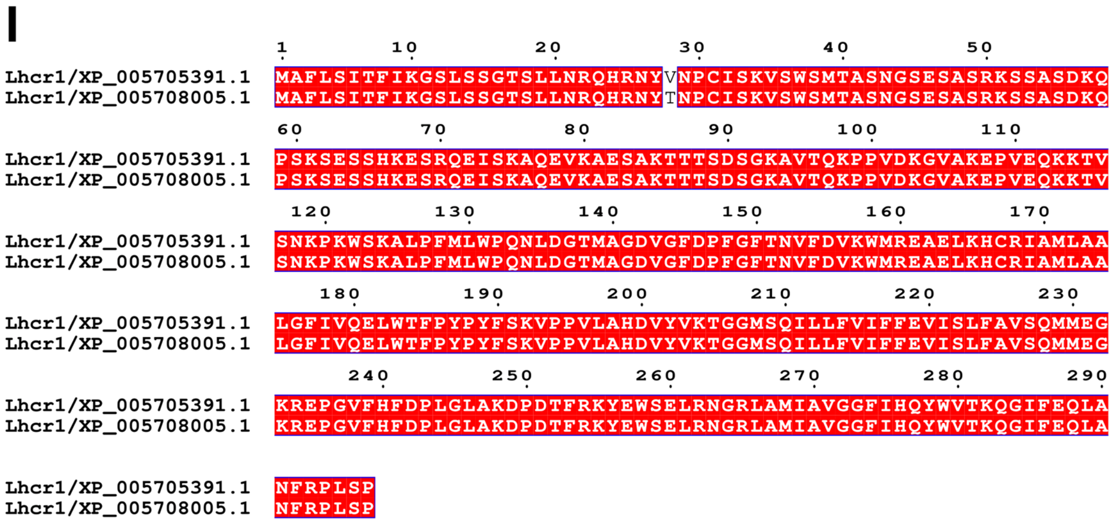

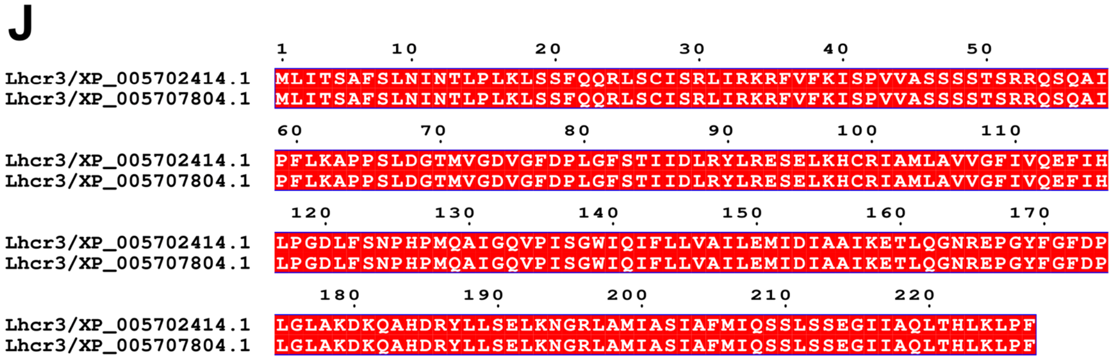

Fig. S9 | Characteristic amino acid residues used for the identification of each LHCI subunit. **A,** Multiple sequence alignment of the LHCI proteins of *G. sulphuraria* NIES-3638 using ClustalW (https://www.genome.jp/tools-bin/clustalw) and ESPript (https://espript.ibcp.fr/ESPript/cgi-bin/ESPript.cgi). Unique residues are indicated by arrows with different colors, which were used for the identification of the LHCI subunits. **B–H,** Characteristic maps and amino acid residues of LHCI-1 (**B**), LHCI-2 (**C**), LHCI-3 (**D**), LHCI-4 (**E**), LHCI-5 (**F**), LHCI-6 (**G**), and LHCI-7 (**H**). The densities and models are shown as meshes and sticks, respectively. The characteristic amino acids are labeled with arrows of the same color as shown in panel **A**. **I, J,** Sequence alignments of two copies of Lhcr1 (**I**) and Lhcr3 (**J**), along with their accession IDs, using ClustalW and ESPript.

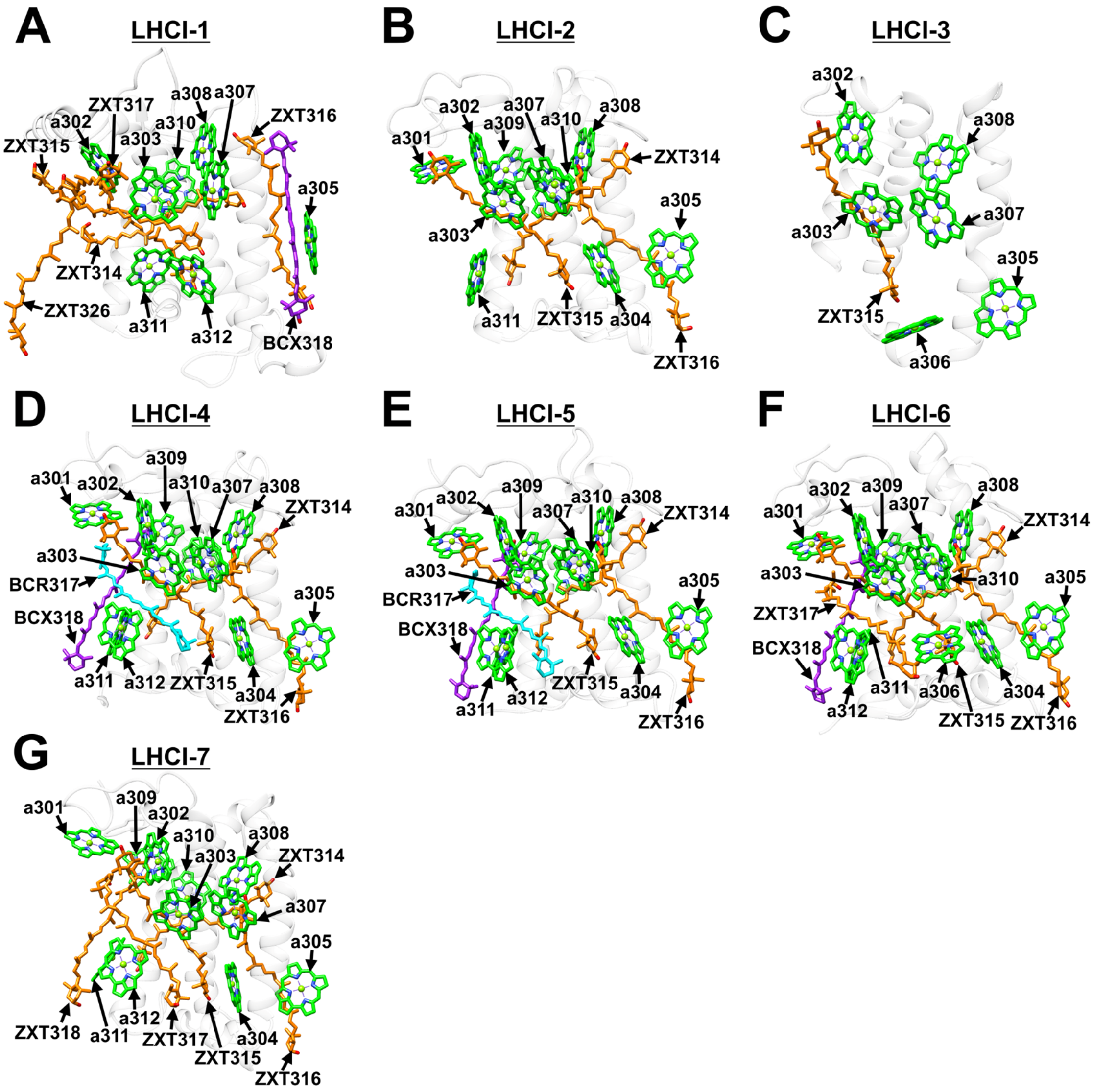

Fig. S10 | Structures of the *G. sulphuraria* LHCIs.

A–G, Structures of LHCI-1 to LHCI-7 depicted as transparent cartoons for proteins and sticks for Chls and Cars with different colors. Only rings of the Chl molecules are depicted. Green, Chl *a*; orange, ZXT; cyan, BCR; purple, BCX.

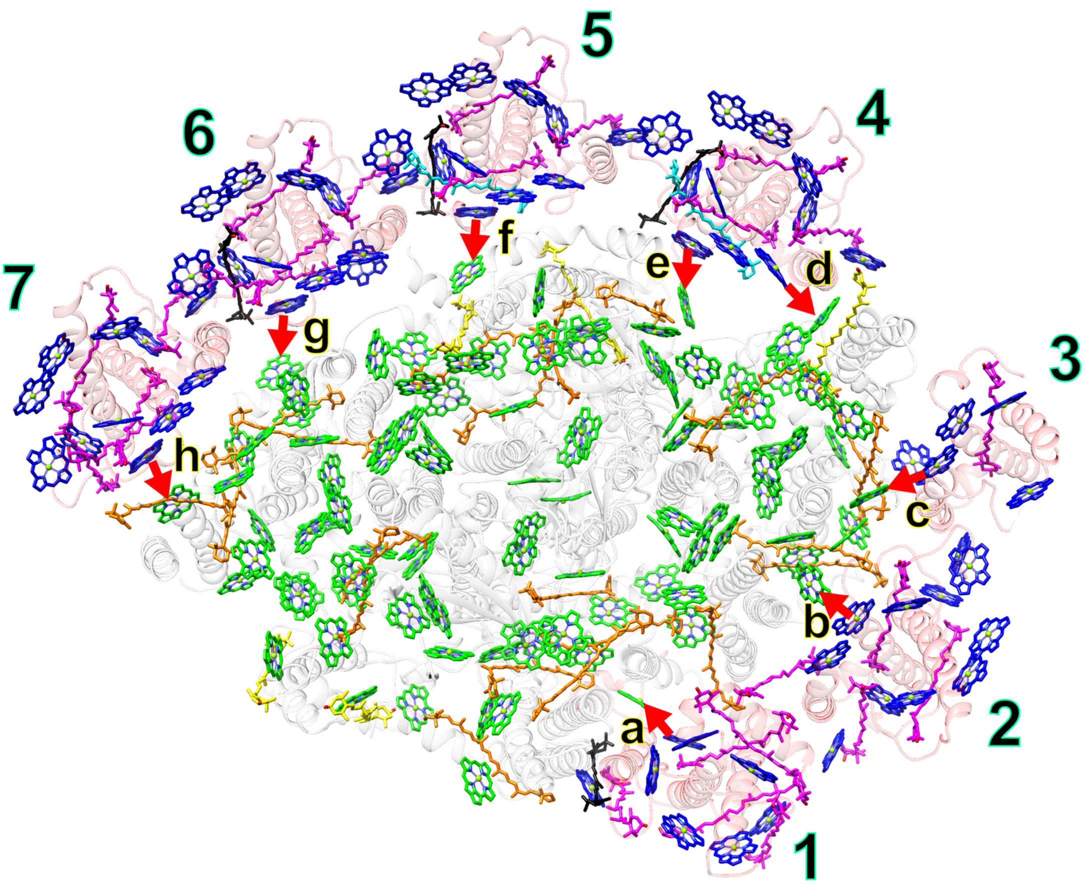

Fig. S11 | Arrangement of pigment molecules within the *G. sulphuraria* PSI-LHCI and possible excitation-energy-transfer pathways from LHCIs to PSI core.

The structure is viewed from the stromal side. The protein structures of PSI core and LHCIs are displayed in transparent cartoons and colored grey and red, respectively. The numbers 1–7 correspond to LHCI-1 to LHCI-7, respectively. Chls and Cars are shown as sticks, and only rings of the Chl molecules are depicted. Green, Chls *a* in PSI; orange, BCRs in PSI; yellow, ZXTs in PSI; blue, Chls *a* in LHCIs; magenta, ZXTs in LHCIs; cyan, BCRs in LHCIs; black, BCXs. Red arrows labeled with letters a–h indicate possible excitation-energy-transfer pathways based on close physical interactions among Chls (see Main text).

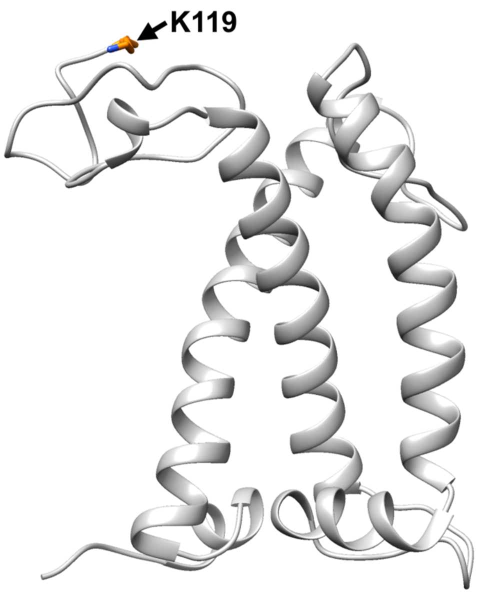

Fig. S12 | Protein structure of the *G. sulphuraria* LHCI-6.
The LHCI-6 subunit is depicted with its protein structure only for clarity (shown as a grey cartoon), starting from K119 (highlighted as an orange stick).

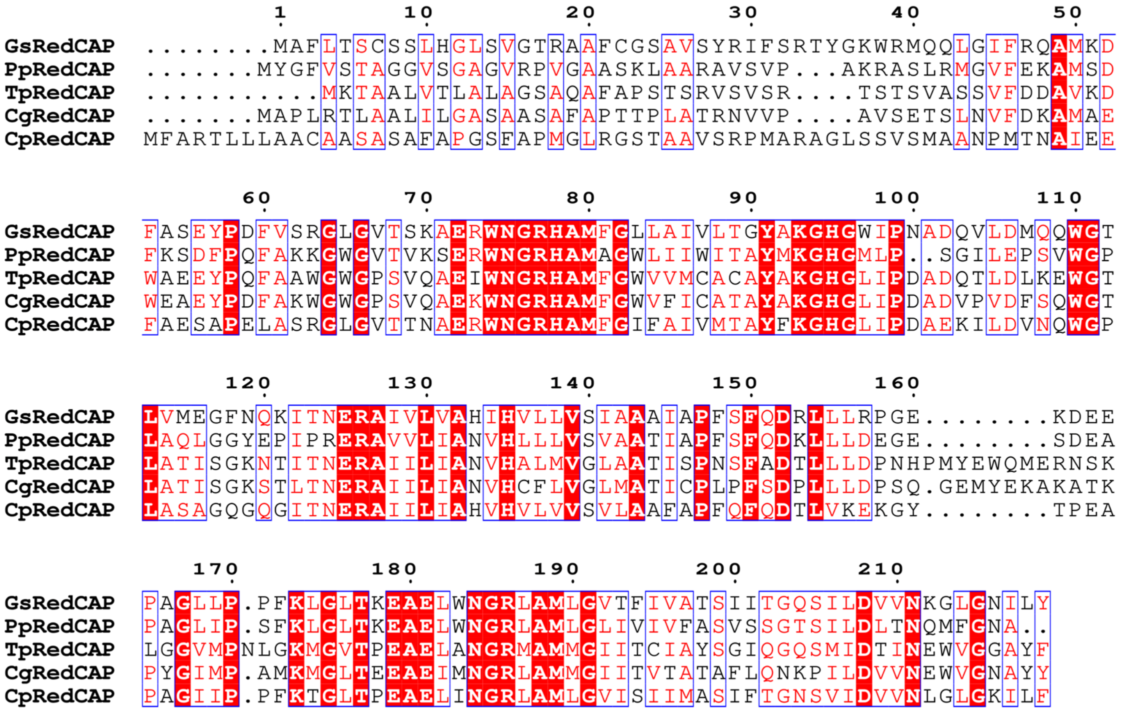

Fig. S13 | Comparison of the amino acid sequences of RedCAPs.

Multiple sequence alignment of RedCAP of *G. sulphuraria* NIES-3638 (GsRedCAP) with those of *P. purpureum* UTEX 2757 (PpRedCAP), *T. pseudonana* CCMP1335 (TpRedCAP), *C. gracilis* UTEX LB 2658 (CgRedCAP), and *C. placoidea* T11 (CpRedCAP) was performed using ClustalW and ESPript.

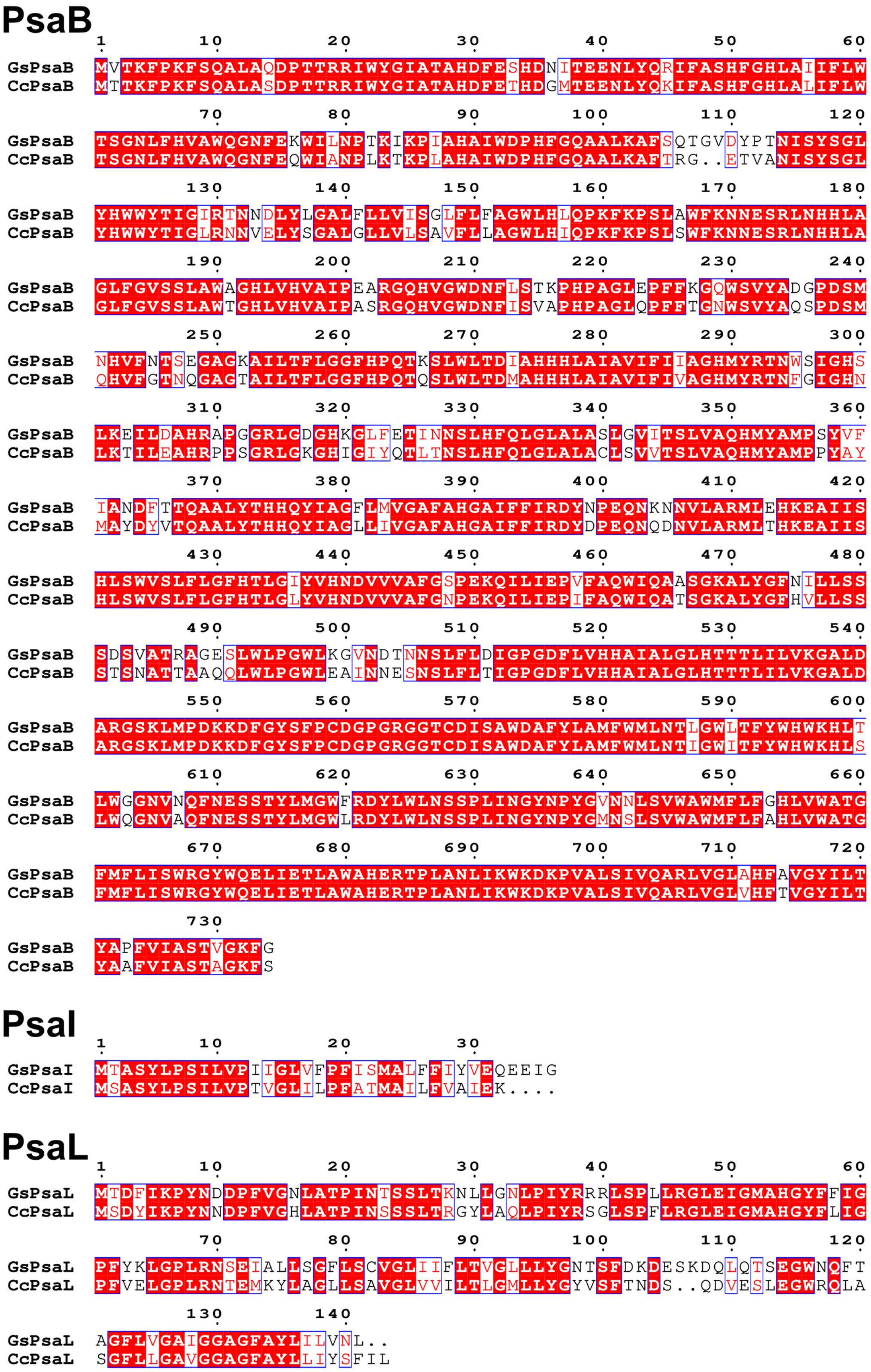

Fig. S14 | Comparisons of the amino acid sequences of PsaB, PsaI, and PsaL.
Each protein sequence was aligned using ClustalW and ESPript: PsaB of *G. sulphuraria* NIES-3638 (GsPsaB) vs. PsaB of *C. caldarium* NIES-2137 (CcPsaB); GsPsaI vs. CcPsaI; GsPsaL vs. CcPsaL.

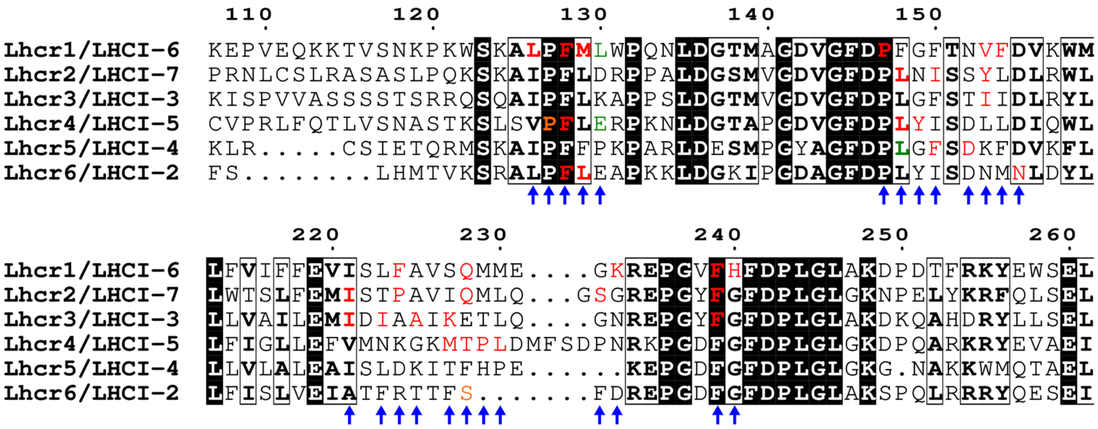

Fig. S15 | Characteristic amino acid residues involved in protein-protein interactions among LHCIs.

Multiple sequence alignment of Lhcrs from *G. sulphuraria* NIES-3638 using ClustalW and ESPript. Each sequence corresponds to gene products (Lhcrs) or subunits (LHCIs), such as Lhcr1/LHCI-6 as summarized in Figure 1. Amino acid residues involved in protein-protein interactions are shown in red (side chains), orange (backbones), or green (both), with their positions marked by blue arrows.

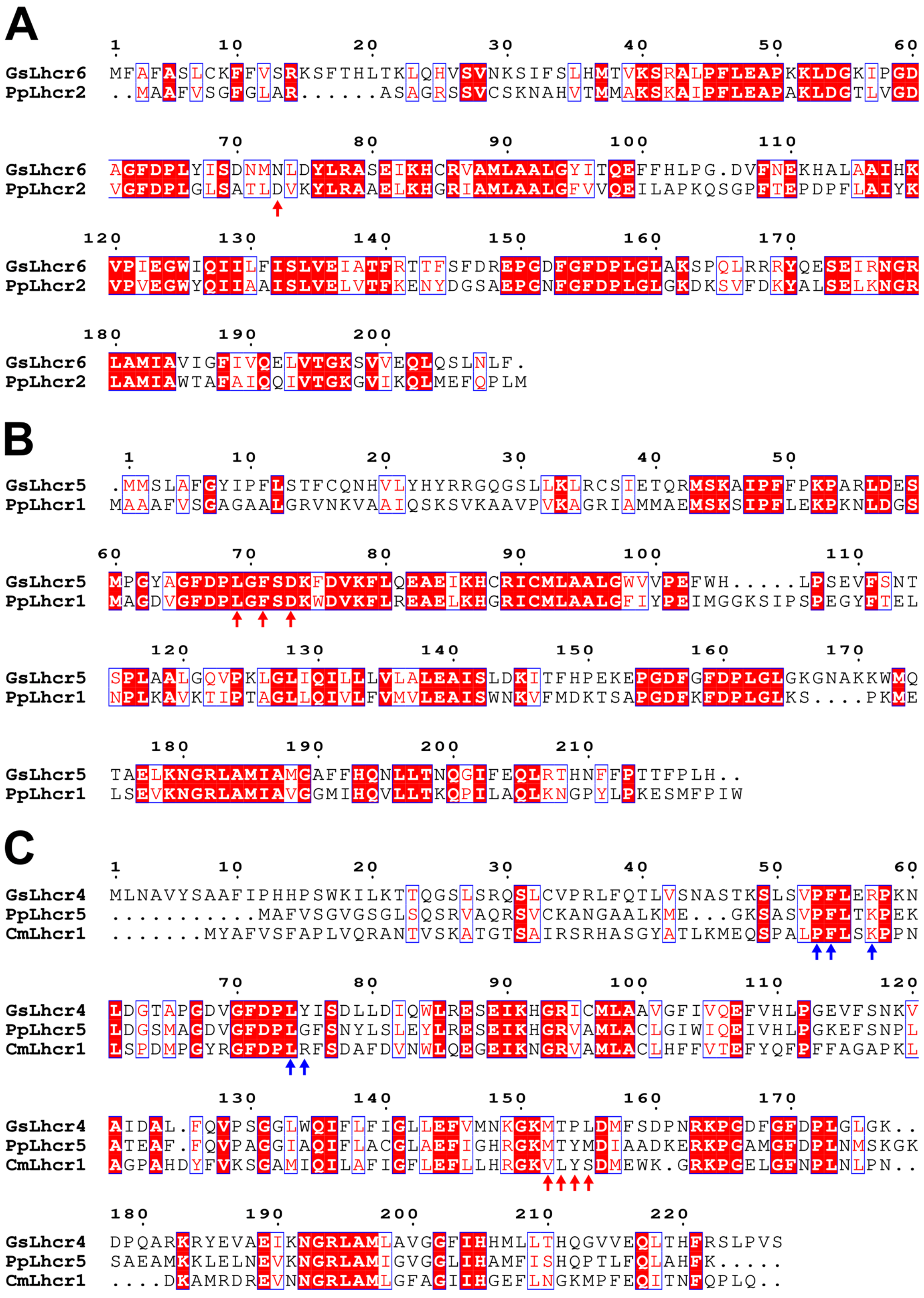
A
GsLhcr6 MFAFASLCKFFVSRKSFTHLTKLQHVSVNKSIFSLHMTVKSRALPFLEAPKKLDGKIPGD
PpLhcr2 ..MAAFVSGFGLAR......ASAGRSSVCSKNAHVTMMAKSKAIPFLEAPAKLDGTLVGD
GsLhcr6 AGFDPLYISDNMNLDYLRASEIKHCRVAMLAALGYITQEFFHLPG.DVFNEKHALAAIHK
PpLhcr2 VGFDPLGLSATLDVKYLRAAELKHGRIAMLAALGFVVQEILAPKQSGPFTEPDPFLAIYK
GsLhcr6 VPIEGWIQIILFISLVEIATFRTTFSFDREPGDFGFDPLGLAKSPQLRRRYQESEIRNGR
PpLhcr2 VPVEGWYQIIAAISLVELVTFKENYDGSAEPGNFGFDPLGLGKDKSVFDKYALSELKNGR
GsLhcr6 LAMIAVIGFIVQELVTGKSVVEQLQSLNLF.
PpLhcr2 LAMIAWTAFAIQQIVTGKGVIKQLMEFQPLM
B
GsLhcr5 .MMSLAFGYIPFLSTFCQNHVLYHYRRGQGSLLKLRCSIETQRMSKAIPFFPKPARLDES
PpLhcr1 MAAAFVSGAGAALGRVNKVAAIQSKSVKAAVPVKAGRIAMMAEMSKSIPFLEKPKNLDGS
GsLhcr5 MPGYAGFDPLGFSDKFDVKFLQEAEIKHCRICMLAALGWVVPEFWH.....LPSEVFSNT
PpLhcr1 MAGDVGFDPLGFSDKWDVKFLREAELKHGRICMLAALGFIYPEIMGGKSIPSPEGYFTEL
GsLhcr5 SPLAALGQVPKLGLIQILLLVLALEAISLDKITFHPEKEPGDFGFDPLGLGKGNAKKWMQ
PpLhcr1 NPLKAVKTIPTAGLLQIVLFVMVLEAISWNKVFMDKTSAPGDFKFDPLGLKS....PKME
GsLhcr5 TAELKNGRLAMIAMGAFFHQNLLTNQGIFEQLRTHNFFPTTFPLH..
PpLhcr1 LSEVKNGRLAMIAVGGMIHQVLLTKQPILAQLKNGPYLPKESMFPIW
C
GsLhcr4 MLNAVYSAAFIPHHPSWKILKTTQGSLSRQSLCVPRLFQTLVSNASTKSLSVPFLERPKN
PpLhcr5 ...........MAFVSGVGSGLSQSRVAQRSVCKANGAALKME...GKSASVPFLTKPEK
CmLhcr1 .......MYAFVSFAPLVQRANTVSKATGTSAIRSRHASGYATLKMEQSPALPFLSKPPN
GsLhcr4 LDGTAPGDVGFDPLYISDLLDIQWLRESEIKHGRICMLAAVGFIVQEFVHLPGEVFSNKV
PpLhcr5 LDGSMAGDVGFDPLGFSNYLSLEYLRESEIKHGRVAMLACLGIWIQEIVHLPGKEFSNPL
CmLhcr1 LSPDMPGYRGFDPLRFSDAFDVNWLQEGEIKNGRVAMLACLHFFVTEFYQFPFFAGAPKL
GsLhcr4 AIDAL.FQVPSGGLWQIFLFIGLLEFVMNKGKMTPLDMFSDPNRKPGDFGFDPLGLGK..
PpLhcr5 ATEAF.FQVPAGGIAQIFLACGLAEFIGHRGKMTYMDIAADKERKPGAMGFDPLNMSKGK
CmLhcr1 AGPAHDYFVKSGAMIQILAFIGFLEFLLHRGKVLYSDMEWK.GRKPGELGFNPLNLPN..
GsLhcr4 DPQARKRYEVAEIKNGRLAMLAVGGFIHHMLLTHQGVVEQLTHFRSLPVS
PpLhcr5 SAEAMKKLELNEVKNGRLAMIGVGGLIHAMFISHQPTLFQLAHFK.....
CmLhcr1 ....DKAMRDREVNNGRLAMLGFAGIIHGEFLNGKMPFEQITNFQPLQ..

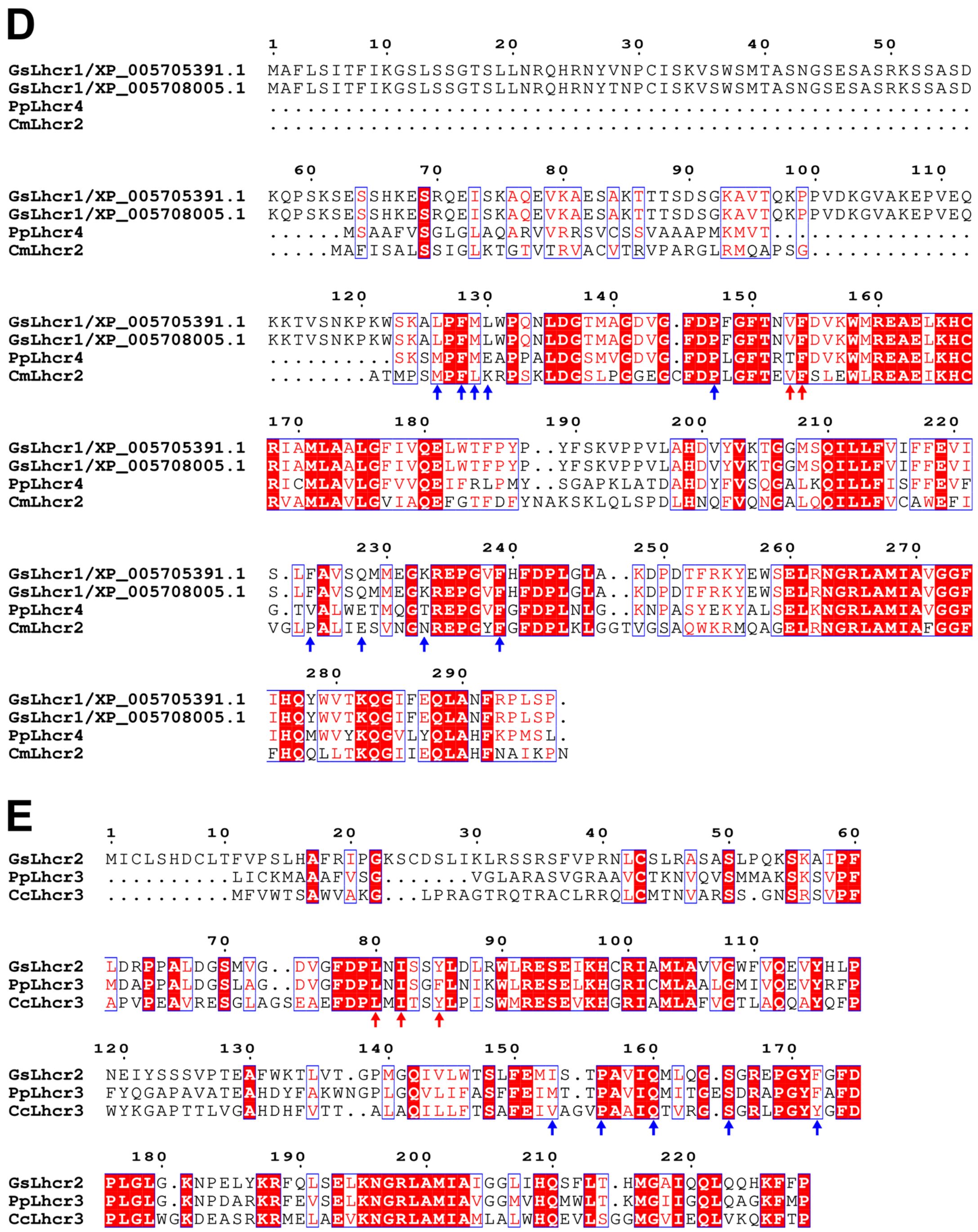

Fig. S16 | Multiple sequence alignments of Lhcrs.

A–E, Alignments of Lhcrs at the LHCI-2 and 4–7 sites, respectively, using ClustalW and ESPript, as summarized in Fig. 6. Gs, *G. sulphuraria*; Pp, *P. purpureum*; Cm, *C. merolae*. Red and blue arrows indicate amino acid residues participating in protein-protein interactions between LHCI and PSI subunits (red) and among LHCI subunits (blue) in the *G. sulphuraria* PSI-LHCI structure, as shown in Fig. 4, 5.

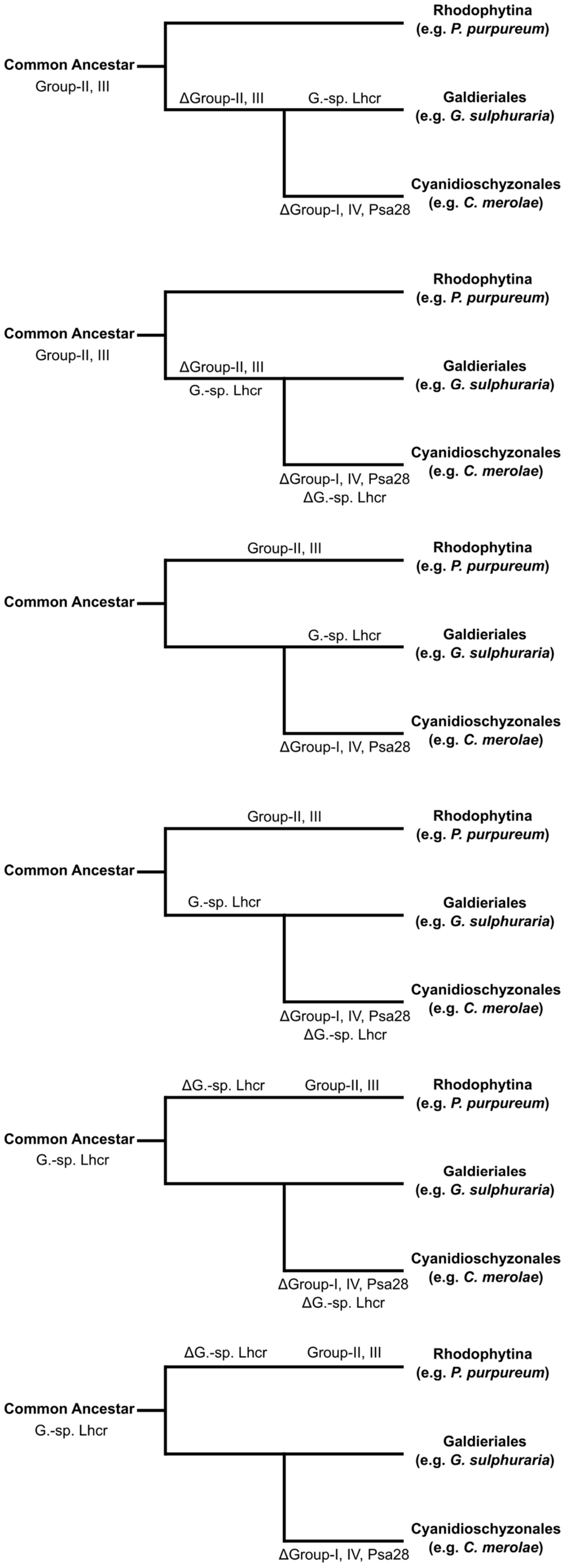

Fig. S17 | Plausible scenarios of the evolutionary trajectory of LHCI subunits.

Six potential scenarios of LHCI acquisitions and losses are depicted on the simplified phylogenetic tree of red algae. G.-sp. in the figure represents Galdieriales-specific Lhcr. Nodes where Lhcr groups and Psa28 either with or without Δ are marked indicate points of loss or acquisition, respectively.

Table S1 | Cryo-EM data collection and structural analysis statistics.

Complex	PSI-LHCI
PDB ID	9KC5
EMDB ID	EMD-62242
Data collection and processing	
Magnification	60000
Voltage (kV)	300
Electron exposure (e^{-}/Å)	50
Defocus range (μm)	−1.8 to −1.2
Pixel size (Å)	0.752
Symmetry imposed	C1
Initial particle images (no.)	2,583,694
Final particle images (no.)	110,313
Map resolution (Å)	2.19
FSC threshold	0.143
Refinement	
Initial Model used	De novo model building
Model resolution (Å)	2.11
FSC threshold	0.5
Map sharpening B factor ($Å^2$)	−43.4
Model composition	
Non-hydrogen atoms	41,435
Protein residues	3,527
Ligand molecules	298
Water molecules	608
B factors ($Å^2$)	
Protein	67.7
Ligand	79.9
Water	52.2
R.m.s deviations	
Bond lengths (Å)	0.028
Bond angles (°)	2.97
Validation	
MolProbity score	1.77
Clashscore	4.24
Poor rotamers (%)	2.98
EMRinger score	6.21
Ramachandran plot	
Favored (%)	96.75
Allowed (%)	2.73
Disallowed (%)	0.52

Table S2 | Averaged *Q*-scores in each subunit.

Subunit	Averaged *Q*-score	
	Postprocessed map	Denoised map
PsaA	0.86	0.86
PsaB	0.86	0.86
PsaC	0.89	0.87
PsaD	0.83	0.83
PsaE	0.80	0.81
PsaF	0.84	0.84
PsaI	0.81	0.82
PsaJ	0.80	0.82
PsaK	0.76	0.79
PsaL	0.79	0.81
PsaM	0.78	0.80
PsaO	0.77	0.80
Psa28	0.81	0.82
LHCI-1	0.69	0.76
LHCI-2	0.62	0.71
LHCI-3	0.50	0.63
LHCI-4	0.77	0.80
LHCI-5	0.76	0.80
LHCI-6	0.76	0.79
LHCI-7	0.56	0.67

Table S3 | Cofactors assigned in each subunit of the PSI-LHCI structure.

Protein	Chlorophyll	Carotenoid	Lipid	Other
PsaA	39 Chl *a*<br>1 Chl *a′*	4 BCR	2 LHG	1 [4Fe-4S] cluster,<br>1 ubiquinone-4
PsaB	42 Chl *a*	8 BCR<br>1 ZXT	1 LHG<br>1 DGD	1 ubiquinone-4
PsaC	-	-	-	2 [4Fe-4S] cluster
PsaD	-	-	-	-
PsaE	-	-	-	-
PsaF	3 Chl *a*	1 BCR	-	-
PsaI	1 Chl *a*	1 BCR	-	-
PsaJ	3 Chl *a*	1 BCR<br>1 ZXT	2 LHG	-
PsaK	2 Chl *a*	2 BCR	-	-
PsaL	3 Chl *a*	3 BCR	-	-
PsaM	-	-	-	-
PsaO	4 Chl *a*	1 BCR<br>2 ZXT		
Psa28	1 Chl *a*	1 BCR<br>1 ZXT	-	-
LHCI-1	8 Chl *a*	6 ZXT<br>1 BCX	1 LHG	-
LHCI-2	10 Chl *a*	3 ZXT	1 LHG	-
LHCI-3	6 Chl *a*	1 ZXT	-	-
LHCI-4	11 Chl *a*	1 BCR<br>3 ZXT<br>1 BCX	1 LHG	-
LHCI-5	11 Chl *a*	1 BCR<br>3 ZXT<br>1 BCX	1 LHG	
LHCI-6	12 Chl *a*	4 ZXT<br>1 BCX	1 LHG	
LHCI-7	11 Chl *a*	5 ZXT	-	-
Total	168	58	11	5

BCR, *β*-carotene; ZXT, zeaxanthin; BCX, *β*-cryptoxanthin; Chl *a*, chlorophyll *a*; Chl *a′*, chlorophyll *a* epimer; DGD, digalactosyl diacyl glycerol; LHG, dipalmitoyl phosphatidyl glycerol.

Table S4 | LHCI proteins identified in the PSI-LHCI structure and their RMSD values with LHCI-4

Protein	Gene	RMSD (Å)/Aligned Cα atoms
LHCI-1	*RedCAP*	3.35/96
LHCI-2	*Lhcr6*	1.08/161
LHCI-3	*Lhcr3*	0.99/93
LHCI-4	*Lhcr5*	0.00/178
LHCI-5	*Lhcr4*	1.48/167
LHCI-6	*Lhcr1*	1.41/167
LHCI-7	*Lhcr2*	1.26/161

Table S5 | Chls and their ligands in each of the LHCI subunits.

Protein	Chlorophyll/ligand
LHCI-1	a302/E72, a303/N75, a305/H135, a307/H78, a308/E181, a310/N184, a311/G213, a312/w1431[2]
LHCI-2	a301/A43, a302/E81, a303/H84, a304/Q98, a305/Q127, a307/E136, a308/E174, a309/LHG320, a310/N177, a311/Q191
LHCI-3	a302/E95, a303/H98, a305/Q141, a306/-[1], a307/E150, a308/E191
LHCI-4	a301/A46, a302/E84, a303/H87, a304/w331[2], a305/Q130, a307/E139, a308/E177, a309/LHG320, a310/N180, a311/Q194, a312/H193
LHCI-5	a301/S51, a302/E89, a303/H92, a304/Q106, a305/Q135, a307/E144, a308/E189, a309/LHG320, a310/N192, a311/H206, a312/H205
LHCI-6	a301/A125, a302/E163, a303/H166, a304/Q180, a305/Q210, a306/H199, a307/E219, a308/E260, a309/LHG320, a310/N263, a311/Q277, a312/H276
LHCI-7	a301/A57, a302/E95, a303/H98, a304/Q112, a305/Q142, a307/E151, a308/E193, a309/-[1], a310/N196, a311/Q210, a312/H209

[1]The ligands of Chls may be water or lipid molecules which cannot be identified due to weak densities.

[2]Water molecules.

Table S6 | Correspondence of numbering of pigments in each PSI subunit described in the text with those in the PDB file.

	PsaB	PsaF	PsaI	PsaO	Psa28
**Chls in the text**	**PDB No. (Chain ID)**	**PDB No. (Chain ID)**	**PDB No. (Chain ID)**	**PDB No. (Chain ID)**	**PDB No. (Chain ID)**
102			301 (1)*		
201					204 (Z)
**Cars in the text**					
202					205 (Z)
203					206 (Z)
206				207 (O)	
207				208 (O)	
304		207 (F)			
853	846 (B)				

*Chain in the adjacent unit.

Table S7 | Correspondence of numbering of pigments in each LHCI subunit described in the text with those in the PDB file.

	LHCI-1	LHCI-2	LHCI-3	LHCI-4	LHCI-5	LHCI-6	LHCI-7
**Chls in the text**	**PDB No. (Chain ID)**	**PDB No. (Chain ID)**	**PDB No. (Chain ID)**	**PDB No. (Chain ID)**	**PDB No. (Chain ID)**	**PDB No. (Chain ID)**	**PDB No. (Chain ID)**
301		302 (2)		302 (4)		304 (6)	303 (7)
302		303 (2)	301 (3)	303 (4)		305 (6)	304 (7)
303		304 (2)	302 (3)	304 (4)		306 (6)	305 (7)
304		305 (2)		305 (4)		307 (6)	306 (7)
305	304 (1)	306 (2)	303 (3)	306 (4)		308 (6)	307 (7)
306			852 (B)*			309 (6)	
307	305 (1)		304 (3)		306 (5)	310 (6)	308 (7)
308	306 (1)		305 (3)		307 (5)	311 (6)	309 (7)
309					308 (5)	312 (6)	310 (7)
310	307 (1)				309 (5)	313 (6)	311 (7)
311	308 (1)				310 (5)	314 (6)	312 (7)
312	309 (1)				311 (5)	315 (6)	313 (7)
**Cars in the text**							
314	310 (1)	312 (2)		313 (4)	312 (5)	316 (6)	
315	311 (1)	313 (2)	306 (3)	314 (4)	313 (5)	317 (6)	
316	312 (1)	314 (2)		315 (4)	314 (5)	321 (5)*	
317	313 (1)			316 (4)	315 (5)	318 (6)	
318	314 (1)			317 (4)	302 (6)*	319 (6)	
319	315 (1)						
326	102 (M)*						

*Chain in the adjacent unit.

Table S8 | Correspondence of numbering of water molecules described in the text with those in the PDB file.

	**PsaI**	**LHCI-4**	
**Chls in the text**	**PDB No. (Chain ID)**	**PDB No. (Chain ID)**	**PDB No. (Chain ID)**
201	203 (I)		
331		401 (4)	
1431			401 (1)
